# A neutral invertase controls cell division besides hydrolysis of sucrose for nutrition during germination and seed setting in rice

**DOI:** 10.1016/j.isci.2024.110217

**Published:** 2024-06-08

**Authors:** Zizhang Wang, Hao Li, Yuxiang Weng

**Affiliations:** 1 Key Laboratory of Plant Molecular Physiology, Institute of Botany, Chinese Academy of Sciences, Beijing 100093, China; 2 Laboratory of Soft Matter Physics, Institute of Physics, Chinese Academy of Sciences, Beijing 100190, China

**Keywords:** Cell biology, Plant biology, Plant development

## Abstract

Sucrose is the transport form of carbohydrate in plants serving as signal molecule besides nutrition, but the signaling is elusive. Here, neutral invertase 8 (OsNIN8) mutated at G461R into OsNIN8m, which increased its charge and hydrophobicity, decreased hydrolysis of sucrose to 13% and firmer binding to sucrose than the wildtype. This caused downstream metabolites and energy accumulation forming overnutrition. Paradoxically, division of subinitials in longitudinal cell lineages was only about 15 times but more than 100 times in wildtype, resulting in short radicle. Further, mutation of OsNIN8 into deficiency of hydrolysis but maintenance of sucrose binding allowed cell division until ran out of energy showing the association but not hydrolysis gave the signal. Chemically, sucrose binding to OsNIN8 was exothermic but to OsNIN8m was endothermic. Therefore, OsNIN8m lost the signal function owing to change of thermodynamic state. So, OsNIN8 sensed sucrose for cell division besides hydrolyzed sucrose.

## Highlights

• OsNIN8 binds to and hydrolyzes sucrose for cell division in root tip during germination
• OsNIN8^Wildtype^ exothermic association with sucrose initiates signal for cell division
• The signal controls cell division of subinitials in longitudinal cell lineages
• Sucrose roles of nutrition and signal can be separated in different mutants

## Graphical abstract

**Figure:**
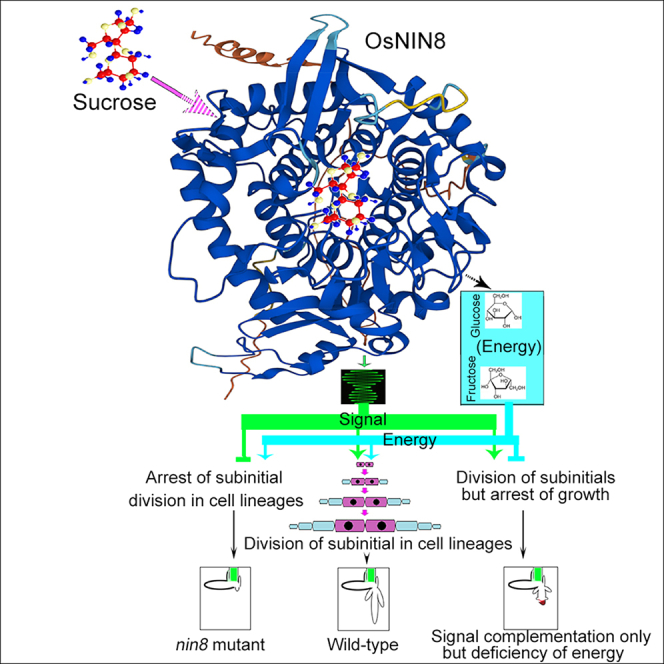

## Introduction

Energetic launching is crucial for seed germination to come up out of the ground in plants. Starch in endosperm degrades into sucrose, maltose, glucose, fructose, and maltotriose by amylases.^1^^,^^2^ These sugars are transported to scutellum, where they are converted to sucrose.^3^ Sucrose is mobilized along phloem and transported to tips of shoot and root via symplastic pathway.^4^^,^^5^ In phloem, sucrose but not glucose or fructose is transported.^6^

In root apical cells, plasmodesms is forming and the plasmodesmata density is increasing during the cell plates are laid down during cell division,^7^ where plasmodesms carry the flux of sucrose and other molecules into newly generated cells as nutrition sources.^8^

Catabolically, invertases hydrolyze sucrose into glucose and fructose. There are three forms of invertase in higher plant. Cell wall invertase (CWinv) and vacuolar invertase (Vinv) are evolutionary relevant but they are not relevant to cytoplasmic invertases (Cinv) in sequence. In addition, CWinvs and Vinvs are acid invertase but Cinvs are neutral/alkaline invertase,^9^^,^^10^ and Cinvs can be separated into neutral or alkaline invertases according to their reactive optimum pH.^11^^,^^12^^,^^13^^,^^14^^,^^15^

It is well known that CWinvs are involved in carbon partitioning, and flower, seed and fruit developments while Vinvs are essential for sugar accumulation and cell expansion, but function of Cinvs remain elusive.^10^^,^^16^ Interestingly, Cinvs are unique to cyanobacteria, such as *Anabaena* inv A and inv B, and plants.^17^ In addition, Cinvs always have more isoforms than CWinvs or Vinvs, and are clustered into α- and β-groups,^9^^,^^10^ for example there are 8 Cinvs in rice.

Mutation of Cinv 8 appears short root during germination and partial sterility in rice.^18^^,^^19^ Similar phenotypes also appear in double mutation of two Cinvs in *Arabidopsis*.^20^ However, these phenotypes only appear from β-group but not α-group of Cinvs in *Lotus japonicus*.^21^ So, it indicates that some Cinvs involve in radicle growth and reproductive development.

Sensing and utilization of available sugar sources are of vital importance for life. Hexokinase (HXK), the first enzyme in the hexose assimilation pathway, is a glucose sensor for plant growth and development in *Arabidopsis*^22^ and OsHXK5 and OsHXK6 are glucose sensors in rice.^23^ However, there are multiple glucose signal pathways involving in diverse genes and processes during response to glucose^24^ and this glucose sensing does not directly control root meristem activation.^25^ This sensor seems to play more roles in glucose repression of photosynthetic genes.^26^ Glucose status in leaf during photosynthesis is different from sucrose status in long distance transport during germination, this sucrose sensing should be different from glucose sensing.

Here, Cinv 8 was cloned by mapping in rice, it was a neutral invertase (OsNIN8) with low *Km* in sucrose hydrolysis. The mutation of a glycine into hydrophobic arginine (OsNIN8m) resulted in a compacter structure and firmer binding to sucrose, which blocked the initiation of sucrose signal, and arrested cell division in radicle during germination and impaired seed setting.

## Results

### Mutation of a neutral/alkaline invertase retards growth of radicle and panicle development

**Figure 1:**
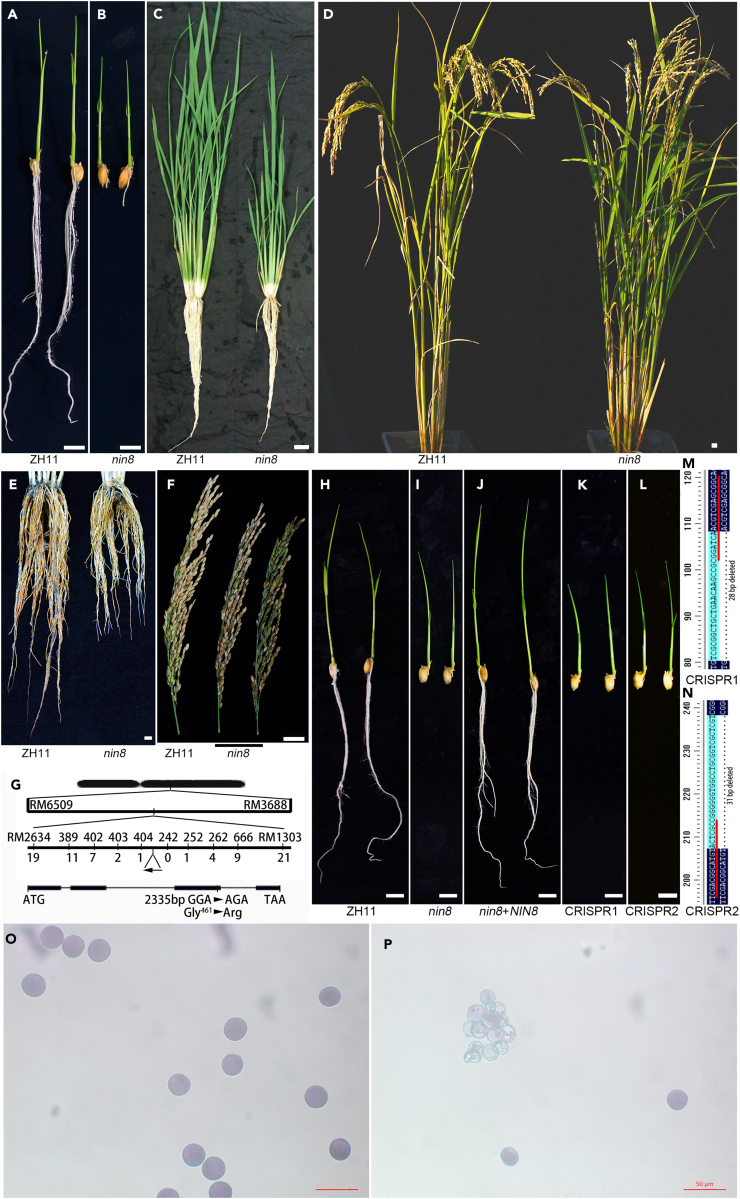
Mutation of an invertase causes arrests of radicle and seed setting (A–D) Phenotypes of WT (ZH11) and mutant (*nin8*) at 10 days after inoculation (DAI) in test-tube culture (A-B), and tillering stage (C) and maturation stage (D) in the field. (E) Root systems of ZH11 and *nin8* at maturation stage, plants were grown in barrel singly and barrels were submerged in the field during the whole growing period, root system was recovered by washing carefully. (F) Mature spikes of ZH11 and *nin8* showing different seed settings in *nin8* spikes. (G) Diagram of map-based cloning, *OsNIN8m* locus was mapped to chromosome 2 between makers 404 and 252. Structure of gene indicated position of nucleotide substitution and amino acid replacement. (H–L) Radicle phenotypes of ZH11 (genotype, *OsNIN8*, H), *nin8* (*OsNIN8m*, I), complementation (*OsNIN8m*+*OsNIN8*, J) and CRISPR/Cas9 knockout (OsNIN8CRISPR, K–L) lines at 10 DAI in test-tube culture. CRISPR 1 and 2, two targets of the knockout. Bar = 20 cm. (M and N) Alignments of target region between ZH11 and knockout lines showing deletions of 28 bp in CRISPR 1 and 31 bp in CRISPR 2. Regions underlined in red were guide sequences. (O and P) Morphology of mature pollen grains in ZH11 (O) and *nin8* (P) by Alexander staining showing most pollen grains were not filling and agglomerated in lighter color in *nin8*. Bar = 50 μm. See also Figures S1–S6 and Table S1.

A mutant showing retarded growth of radicles was obtained in a screen on progeny population of sodium azide treatment for root phenotypes, exhibiting short or bald root during germination in rice (*Oryza sativa* L. subsp. *Japonica* cv. ZH11). Its shoot was slightly stunted but development rate unchanged (Figures 1A and 1B). The dwarfism was relieved with age or supplementing sugars from photosynthesis (Figure 1C), which was consistent with that the photosynthesis-derived glucose was decoupled from glucose sensing in *Arabidopsis* seedlings during germination.^25^ Finally, the plant height was slightly shorter and the root system was shorter than those in wild type (WT, ZH11), and its rate of seed setting was only 0–31% (Figures 1D–1F).

The segregation ratio between mutant and WT phenotypes of F2 population after crossing with an indica rice, NJ6, was 3:1 (*χc2* = 3.0543, *p* > 0.05), exhibiting its genotype was recessive homozygous single gene. To clone the gene, a map-based cloning strategy was used, the gene was mapped on chromosome 2 near marker RM525 in rough mapping of genomic search (Figure S1) and localized between markers RM2634 and RM1303 after mapping with markers near RM525 from 323 short root seedlings (Figure 1G). Eight markers were developed between genomic sequences of indica cv. 9311 and japonica cv. Nipponbare for fine mapping (Table S1). Finally, the gene was localized between markers 404 and 252 from more than 1200 seedlings, where contained 5 genes. After sequencing, a single nucleotide G at position 2,335 of LOC_Os02g34560 was found being mutated into A, resulted in an arginine residue taking the place of the 461-glycine residue of the protein. The gene codes a neutral/alkaline invertase, designated as *OsNIN8* following a previous catalog.^9^

To confirm the mutation, complementation experiment was conducted via transformation of a 6,654-bp genomic clone of *OsNIN8* including its native 2,409-bp putative promoter and 1,171-bp 3′-UTR into the mutant. And 16 of 17 independent transgenic lines recovered normal root and seed setting (Figures 1H–1J and S2). Furthermore, *OsNIN8* was knocked out involved use of CRISPR/Cas9 technology. Two targets within exon 1 were designed as guide sequences. Cas9-free lines were obtained from backcross progeny. Both knockouts reproduced the mutant phenotypes (Figures 1K–1N).

The low seed setting rate of the mutant was mainly due to pollen abortion, normal pollen grains in ZH11 was 96% but only 11.4% of pollen grains was normal in the mutant (Figures 1O and 1P). And worse, if plants were cultured under cool condition at panicle differentiation stage, e.g., in artificial climate chamber or autumn, plant, panicle, pollen development and seed filling were well in ZH11, but the mutant appeared small and short panicles, little panicle branching, aplasia in glume, stigma, anther and pollen of floral organ, even small anther and no pollen though the growth of plant unchanged (Figures S3A–S3E).

Therefore, both growths of radicles and reproductive organs were restricted in mutation of *OsNIN8* (*OsNIN8m*). Similar phenotype of this gene was observed previously.^18^^,^^19^ This mutant was nominated as *nin8*.

The transcriptional levels of this gene in root at 17 days-after-inoculation (DAI) between *nin8* and WT remained unchanged (Figure S4). Overexpression or knockdown (RNAi) of *OsNIN8* in WT did not change the phenotypes (Figures S2 and S5).

There were 8 alkaline/neutral invertases in rice.^9^ OsNIN8 was evolutionarily separated from the others using a conserved region from 99 to 543 residues of OsNIN8 for alignment (Figure S6A). Interestingly, individual knockout of *OsNIN1-7* had not these phenotypes at all (Figures S6B–S6D). So, it was *OsNIN8* but not *OsNIN1*-*7* played the roles for these phenotypes.

### Retarded growth of radicle is due to a block in subinitial division in *nin8*

**Figure 2:**
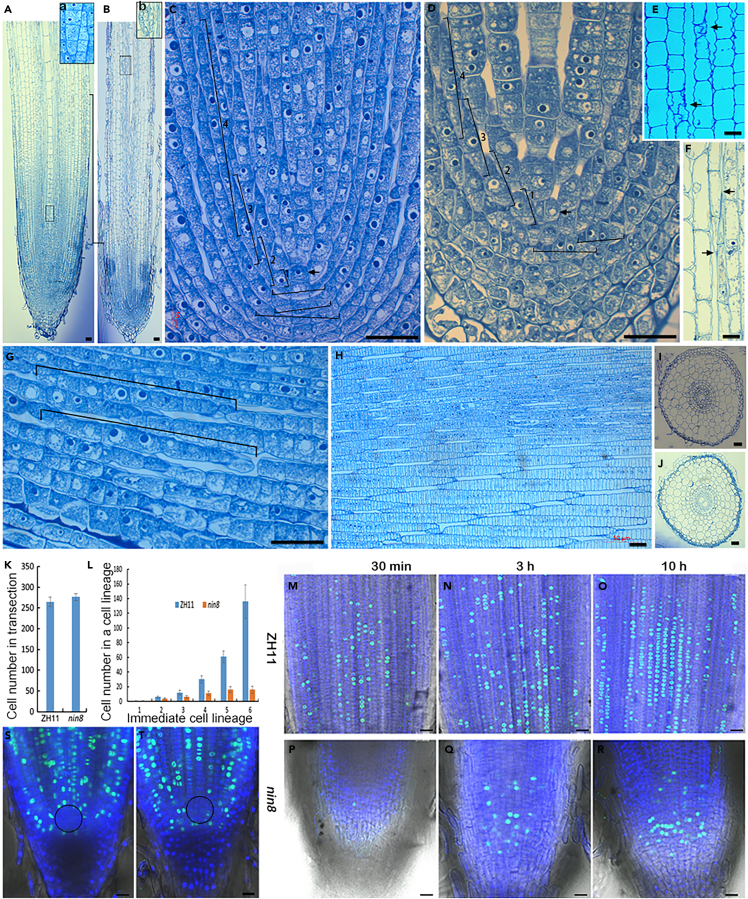
Arrest of subinitial division for elongation of longitudinal cell lineages stunts root growth in *nin8* (A and B) Longitudinal section of ZH11 (A) and *nin8* (B) root tips at 10 DAI stained by toluidine blue O. Boundary lines between elongation and mature regions and distance between two boundary lines were indicated showing the length of dividing regions in ZH11 and *nin8*; a, showing the central procambium cell lineage was composed of different cell lineages (a connection region); b, emergence of lateral root in *nin8* showing the root was short. (C and D) Longitudinal sections of meristematic zone in ZH11 (C) and *nin8* (D). Some cell lineages were indicated with line segments: longitudinal line segments indicated cell lineages in the meristem; horizontal line segments indicated cell lineages in the root cap, number represented age of cell lineages after division from the initial. Arrowheads pointed to initials. Cells with large nucleus indicated cell in mitotically active, two adjacent cells with similarly large nucleus indicated they were latest produced from the same division, cells with nuclei getting smaller and smaller on both flanks alternately indicated they were from earlier divisions; Cavities between cell lineages remained from sizes of cell became larger and larger basipetally but elder cells no longer grew with age forming spindle cell lineages. (E and F) Joints of two cell lineages in *nin8* (E) and ZH11 (F). Arrowheads, ends of cell lineages. (G) Image away from growth cone showing cell lineages clearer in ZH11. Line segments indicated the length of the cell lineage, cell lineages about similar length gathered together indicated their ages were similar. (H) Cell lineages in cortex of root tips in *nin8* at tillering stage showing ordering of cell lineages. (I and J) Transverse sections at maturation zone of radicles in ZH11 (I) and *nin8* (J) showing their cell (cell lineage) numbers, each cell represented a cell lineage divided from the initial. (K) Cell (cell lineage) numbers in ZH11 and *nin8* were similar. Cell number of 6 sections from 2 root-tips each, data were represented as mean ± SEM. (L) Cell numbers from generation 1–6 cell lineages between ZH11 and *nin8* showing a big difference. Generations 1–6 of cell lineage, from the initial basipetally end to end connection cell lineages were defined as different generations. Cell number of 6 cell lineages from sections of 2 root-tips each, data were represented as mean ± SEM. (M–O) EdU incorporation and label of newly replicated DNA. Seedlings of ZH11 at 10 DAI were fed with EdU for 30 min, 3 h and 10 h, and incorporated EdU was detected by immunofluorescence. A queue of cells with EdU labeled DNA indicated newly divided cells during the treatment time in the center of a cell lineage. Scattered cell queues indicated subinitials distributed in meristem and elongation regions everywhere. Lengths of cell queue getting longer and longer successionally with treatment time indicated division of subinitials were in a line manner continually. (P–R) Cells with EdU incorporation in DNA in *nin8* treated with EdU for 30 min, 3 h and 10h. (S and T) Root tips of ZH11 after EdU incorporation for 10 h showing no DNA synthesis in QC regions (circle region). Bar = 20 μm. See also Figure S11.

To explore the retarded growth of radicle in *nin8* we compared semi-thin sections between root tips of *nin8* and ZH11. Region of cell dividing in ZH11 was 760 μm but in *nin8* was 290 μm in length at DAI 10 (Figures 2A and 2B). Interestingly, some cells with heavy nucleus, which indicated that they were in mitotic activity, were always larger than their bilateral cells forming a cell lineage; and there were cavities appeared between cell lineages, so it was easy to distinguish cell lineages by two smaller and smaller ends of a cell lineage in *nin8*. In the contrasting manner, this pattern of cell lineages was also found in ZH11 (Figures 2C and 2D).

Importantly, it could be recognized that an initial located at the origin position of cell division closing to the root cap cells (Figures 2C and 2D). It could be disclosed that the initial carried out transverse division apically to produce root cap subinitials, whose divisions were only in periclinal manner forming horizontal cell lineages to constitute the root cap. Simultaneously, the initial divided periclinally to generate subinitials for root primary meristem, these subinitials divided in transverse manner to form longitudinal cell lineages.

These fusiform cell lineages indicated that cells at symmetric ends divided earlier in the niche when and where cells were small and compacted close to root tip. In pace with the growth of root tip, cells were getting larger basipetally, the newly subinitials were getting larger and larger. So, it emerged that two biggest cells with heavy nucleus (one was subinitial the other was not) in the center of a cell lineage, only the subinitial preserved its subinitial identity and the other lost the potential. Subinitials generated no-subinitials alternately toward both sides of themselves forming fusiform cell lineages to keep subinitials at the center of cell linages. Cells without heavy nucleus bilaterally had lost division activity. The connection of two cell lineages (one end of each) was always forming lapped joint, and there were cavities around the joint since they were the smallest cells in the cell lineage (Figures 2E and 2F).

The paradigm of cell lineage was clearer by examining from looser regions of the root tip, where the smaller ends and the connections were easier to be recognized, in ZH11 (Figure 2G), and from root tips of crown root at tillering stage where cell lineages were more and longer than in radicle in *nin8* (Figure 2H).

Cell size and cell number in transection of radicles between ZH11 and *nin8* were similar (Figures 2I–2K), indicating that division times of initial in ZH11 or *nin8* for number of cell lineages contributing to root thickness were similar.

Tracking these cell lineages basipetally, we could recognize more cell lineages one by one from their connections (ends) (Figures 2C–2H). We designated cell lineages end to end axial connection into different generations. To count cell number in cell lineages of different generations we took images of longitudinal section continuously from root tip to mature zone. In *nin8*, cell number in cell lineages of generation 5 were about 14–17 cells, i.e., their subinitials divided for 14–17 times, and there were no longer more cells after generation 5; but in ZH11, cell number in cell lineages of generation 6 were more than 100 (Figure 2L). Thus, the stunted growth of roots in *nin8* was due to blocking of subinitial division in cell lineages.

To confirm cell division occurred only in the center of cell lineages. Shoots of ZH11 and *nin8* at DAI 10 were treated with EdU solution for 30 min, 3 h and 10 h for EdU label. Cell nuclei incorporated with EdU were imaging and analyses. For ZH11, cells with nucleus of newly synthesized DNA often exhibited two or a few cells adjoining in line and distribution in the meristem and the elongation zones at treated with EdU for 30 min. The cell queues were getting longer and longer in line from 3 h to 10 h treatments forming the middle segments of cell lineages (Figures 2M–2O). Nascent cells with Edu labeled DNA in *nin8* root were little (Figures 2P–2R) indicating cell division was very slow.

Interestingly, cells with newly DNA replication did not gather into but dispersed around the quiescent center (QC) in ZH11 (Figures 2S and 2T), because cells in the QC were all in acropetal flank of cell lineages, where they were far away from subinitials, except nascent subinitials close to the initial. These cells had lost the mitotic activity early after exiting from subinitial identity.

In summary, OsNIN8 controlled the growth of root length was mainly controlled division times of subinitial in longitudinal cell lineages.

### Retarded growth of *nin8* roots is not due to attenuation of sucrose hydrolysis of OsNIN8m

Invertase hydrolyzes sucrose into glucose and fructose, and then glucose enters glycolytic pathway for metabolism. If dwarf root of *nin8* was due to attenuation of sucrose hydrolysis it could be rescued by external glucose, and should be depletion of downstream metabolites such as glucose, fructose, organic acids and amino acids in roots. However, the phenotype of stunted radicle in *nin8* was not recovered by adding glucose or sucrose to medium during germination (Figure S7A). To check osmotic effect on this sugar complementation, equal molar of mannitol was used as control (Figure S7B).

Contents of sucrose, maltose and some metabolites downstream of sucrose were determined in root, shoot and endosperm at DAI 10 and DAI 17 using GC-MS or GC-MS/MS methods (root sample at DAI 10 was missing because of hard to collection; Tables 1 and S2–S7). As comparison with in ZH11, glucose, fructose, citric acid and 14 free amino acids were highly accumulated in root, endosperm and leaf of *nin8* except glutamic acid in root at DAI 17 and some amino acids in leaf at DAI 10 and DAI 17; interestingly, sucrose and maltose were also accumulated in *nin8* (Tables 1 and S2–S7).

**Table 1:** Ratio of sugars, citric acid and amino acids contents in *nin8* to those in WT (Fold change)

	DAI	Suc	Mal	Glc	Fru	Cit	Ala	Val	Leu	Ile	Pro	Gly	Ser	Thr	Asp	Met	Glu	Phe	Lys	Tyr
Root	17	36.96	50.00	347.12	35.05	30.95	10.20	5.85	7.29	6.15	9.58	7.77	8.72	5.05	8.62	2.09	0.90	2.99	3.92	14.45
Endo-sperm	10	2.18	620.88	6.99	6.09	2.10	3.80	7.07	6.01	6.92	7.50	2.83	6.32	3.11	5.59	4.23	1.74	6.95	5.09	20.43
	17	1055.8	4548.1	1415.7	135.75	7.56	5.76	11.44	19.94	15.70	14.34	13.00	21.34	7.00	10.40	4.72	2.32	8.57	11.90	–
Leaf	10	24.43	12.82	2.10	5.46	0.58	1.49	3.04	2.75	2.60	2.67	3.34	5.24	2.69	0.69	4.22	0.93	1.13	1.36	1.20
	17	15.01	63.75	2.52	3.42	4.80	4.39	0.36	0.55	0.20	0.87	0.85	1.50	0.61	3.78	0.65	1.18	0.13	1.02	0.54

Root, endosperm and leaf samples of ZH11 and *nin8* at DAI (day after inoculation) 10 and 17 were collected, sugars and citric acid were determined involved use of GC-TOF-MS method and free amino acids were determined use of GC-QqQ-MS/MS method (data were from 3 independent experiments). Data was ratio of content (mean) of a metabolite in *nin8* to in ZH11, data of root at DAI 10 was missing owing to hard to collect roots from *nin8* then. Suc, sucrose; Mal, maltose; Glc, glucose; Fru, fructose; Cit, citric acid; Ala, alanine; Val, valine; Leu, leucine; Ile, isoleucine; Pro, proline; Gly, glycine; Ser, serine; Thr, threonine; Asp, aspartic acid; Met, methionine; Glu, glutamic acid; Phe, phenylalanine; Lys, lysine; Tyr, tyrosine. Raw data saw Tables S2–S7. See also Figure S7 and Tables S2–S7.

In *nin8*, glucose was 6.99 times at DAI 10 and 1415.7 times at DAI 17 in endosperm, 347 times in root at DAI 17, and 2.10 times at DAI 10 and 2.52 times at DAI 17 in leaf of that in WT; Accordingly, fructose were 6.09 times, 135 times in endosperm, 35 times in root, 5.46 times and 3.42 times in leaf; Maltose was 620.88 times and 4548.1 times in endosperm, 50 times in root, 12.82 times and 63.75 times in leaf; Sucrose were 2.18 times and 1055.8 times in endosperm, 36.96 times in root, 24.43 times and 15 times in leaf. Citric acid were 2.1 times and 7.56 times in endosperm, 30.95 times in root, 0.58 times and 4.8 times in leaf; Range of amino acids were 20.43–1.74 times and 21.34–2.32 times in endosperm, 14.45–0.9 times in root, 5.24–0.69 times and 4.39–0.2 times in leaf of those in WT, respectively (Tables 1 and S2–S7).

It made sense that sucrose, the substrate of invertases, accumulated in *nin8*. Because continually upstream flow of sucrose from endosperm during germination and attenuation of hydrolysis by OsNIN8m in *nin8* led to sucrose accumulation. But it was difficult to understand that downstream metabolites such as glucose, fructose, citric acids and amino acids accumulated in *nin8*.

Maltose was the main product of starch degradation in endosperm during germination^1^^,^^2^ and in transitory starch breakdown at night in leaves,^27^ where it was converted into sucrose in the cytosol by amylomaltases,^28^^,^^29^ serving as an intermediate of storage sugar. So, it could accumulate in endosperm, root and shoot during germination.

From DAI 10 to DAI 17, concentrations of most metabolites decreased to levels close to their respective physiological concentration in ZH11 but these decreases were very small in *nin8* (Tables S4–S7). Moreover, accumulations of these metabolites were acuter in endosperms or roots than in leaves, where most amino acids in leaves at DAI 17 in *nin8* were close to levels in ZH11 (Tables S4–S7). It was consistent with that leaves were grew better than roots in *nin8* then.

The highly accumulation but not depletion of sucrose downstream metabolites in *nin8* indicated that *nin8* did not restricted supplies of energy and materials from mutation of *OsNIN8* into *OsNIN8m*. There must be another reason for short roots.

Mutation of a single gene produced contradictory phenotypes of arrest cell division for root growth and overnutrition simultaneously. The only possible situation was that this gene first arrested cell division forming short roots, which stopped nutrition assimilation. Therefore, these metabolites gradually accumulated in cells since sucrose flow from endosperm continued. Nutrient source could not be used on cell division for growth in these cells.

### The sucrose binding site of OsNIN8m is hydrophobic and charge blocked

Alignment of homologs showed that the conserved region of OsNIN8 also conserved across cyanobacteria to other higher plants (Figure S8). Homology modeling (SWISS-MODEL) disclosed that OsNIN8 matching an *Anabaena* alkaline invertase 2.11 Å structure with GMQE = 0.69 and QMEAN = −1.75.^30^ The structure was an (α/α)_6_ barrel with two concentric layers wall (6 α-helixes forming one layer each) specific binding sucrose for hydrolysis inside the barrel and the α-helix_12_ was suspending above the opening of the barrel^30^^,^^31^ (Figure S9A).

**Figure 3:**
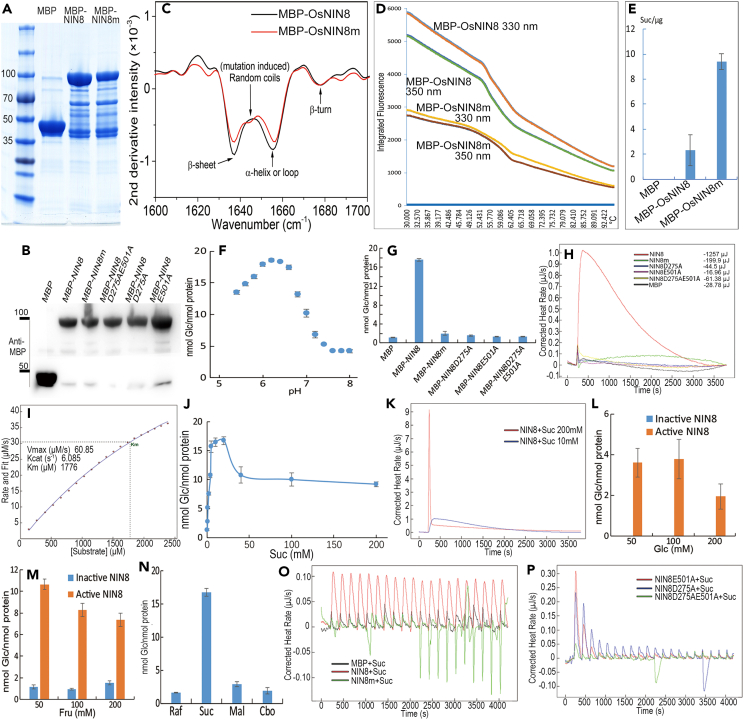
Different mutations of OsNIN8 alter its structure and characteristics to separate sucrose binding from sucrose hydrolysis in enzymology (A and B) Purification of proteins. Proteins were expressed in *E*. *coli*, purified using resin, dialyzed for salt removal and dissolved in 50 mM potassium phosphate buffer (pH6.4); MBP-tag (MBP), MBP fused to OsNIN8 (MBP-OsNIN8) and MBP-OsNIN8m were checked by Coomassie blue staining (A); MBP, MBP-OsNIN8, MBP-OsNIN8m, MBP-OsNIN8D275A, MBP-OsNIN8E501A, and MBP-OsNIN8D275AE501A proteins were checked by immunoblotting with MBP antibody (B). (C) Conformational comparison of MBP-OsNIN8 with MBP-OsNIN8m determined by FTIR spectroscopy assay. A mutation induced random coils at 1645 cm^−1^ on OsNIN8m was indicated. (D) Comparison of protein unfolding between MBP-OsNIN8 and MBP-OsNIN8m using nanoDSF assay. Temperature range, 30°C–95°C (horizontal ordinate). Recording fluorescence at 330 nm and 350 nm showing OsNIN8m was more compact (lower tryptophan fluorescence intensity) than OsNIN8 (*n* = 2). (E) MBP-OsNIN8 and MBP-OsNIN8m affinities to sucrose was determined by pulldown assay using amylose magnetic beads, and sucrose was determined using GC-TOF MS after washing and collection. Data were from 3 independent experiments and represented as mean ± SEM. (F) The optimum pH of OsNIN8 for sucrose hydrolysis into glucose was determined in 50 mM potassium phosphate buffer using microplate assay recorded at 450 nm. Data were from 3 independent experiments and represented as mean ± SEM. (G) Sucrose hydrolysis activities of OsNIN8, OsNIN8m, OsNIN8D275A, OsNIN8E501A and OsNIN8D275AE501A using microplate assay. Data were from 3 independent experiments and represented as mean ± SEM. MBP tag was as the negative control. (H) Enthalpy changes of sucrose hydrolysis of OsNIN8, OsNIN8m, OsNIN8D275A, OsNIN8E501A, OsNIN8D275AE501A and MBP-tag involved using the single injection of ITC assay. Reaction for 1 h at 25°C. Cumulative heat rates were indicated. (I) Enzyme kinetics of OsNIN8 for sucrose hydrolysis characterized by single injection of ITC assay. Parameters of the reaction were indicated. (J and K) High concentrations of sucrose inhibited hydrolysis activity of OsNIN8 determined by microplate (J, data were from 3 independent experiments and represented as mean ± SEM) or ITC (K). *Km* of microplate determine was fit to reaction curve from 0 to 20 mM of series sucrose concentration. (L and M) Concentrations of glucose (L) and fructose (M) did not inhibit OsNIN8 activity. Data were from 3 independent experiments and represented as mean ± SEM. OsNIN8 was boiled to inactivate as negative control, and different glucose or fructose concentrations were added before reaction as background, OsNIN8 increased glucose contents were shown. (N) OsNIN8 specific hydrolyzed sucrose but had little activities for hydrolysis of raffinose, cellobiose or maltose into glucose using microplate assay. Data were from 3 independent experiments and represented as mean ± SEM. (O) Titration of MBP, MBP-OsNIN8 and MBP-OsNIN8m with sucrose using ITC assay. Titration was 20 injections with interval 200 s at 25°C. Peak value > 0, Exothermic; Peak value < 0, endothermic; MBP titrating with sucrose was as negative control, its peaks were the background from the physical motion of titration; Exothermic peaks of OsNIN8 indicated heats of sucrose hydrolysis; Inversion of exothermic to endothermic peaks of OsNIN8m indicated that its endothermic binding to sucrose was offset by exothermic sucrose hydrolysis (it remained 13% activity of hydrolysis) before injection 10, when OsNIN8m binding sucrose reached an equilibrium, after then was net endotherm of binding. (P) Titrations of OsNIN8D275A, OsNIN8E501A and OsNIN8D275AE501A with sucrose. No hydrolyzing exotherm as OsNIN8 shown but the peak heights getting lower and lower indicated the sites for sucrose docking lessening with the titration showing a strong exothermic association between proteins and sucrose. See also Figures S8–S10.

**Figure 4:**
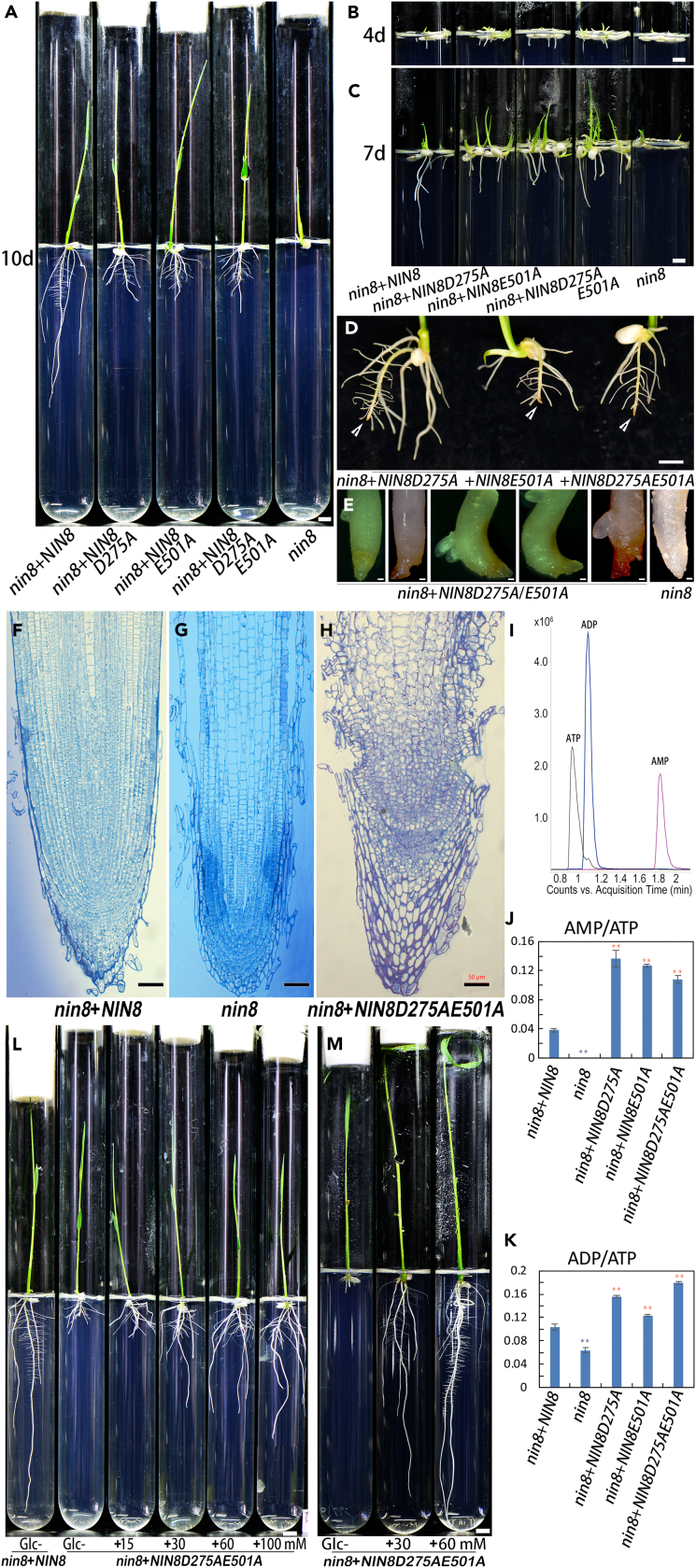
Catalytic mutation of *OsNIN8* separates sucrose signaling from sucrose hydrolysis in cytology (A–C) Germination of mutants deficient in sucrose hydrolysis of OsNIN8. Transgenic lines of complementation of *OsNIN8m* (*nin8*) with *OsNIN8D275A*, *OsNIN8E501A* and *OsNIN8D275AE501A* for deficiency of OsNIN8 hydrolytic activity. Seeds germinated for 4 days (B), 7 days (C) and 10 days (A) were shown. Root length and shoot height were similar to positive control (*nin8*+*OsNIN8*) but were longer than that in negative control (*nin8*) showing normal cell division at DAI 4 indicated signal for cell division was working (B); root lengths only a half at DAI 7 (C) and one-third at DAI 10 to the positive control because their nutrient source was only 13% of the positive control from *nin8*. bar = 6 mm. *nin8* arrested cell division all the time. (D and E) Roots of three catalytic mutation lines stopped growing at about 1 cm in length with root tips turning brown (D) and appeared dysmorphosis after magnification (E) at DAI 10. bar = 6 mm. (F–H) Longitudinal sections exhibited cell division continuing in the meristem but elongation stop forming an inflated meristem and burst in hydrolysis-deficient line (H) as compared to positive (F) and negative (G) controls. bar = 50 μm. (I–K) Catalytic mutation lines were lack of energy. Assay of ATP, ADP and AMP using UPLC-Q-TOF/MS. (I) Peak standards of ATP, ADP and AMP. Ratios of AMP/ATP (J) or ADP/ATP (K) were greater than that in positive control (*nin8*+*OsNIN8*) indicated lack of energy, while *nin8* was rich in energy. Data were from 3 independent experiments and represented as mean ± SEM. Double star in red indicated significant increase; in black indicated significant decrease. (L) Rescue of root growths for a catalytic mutation line by addition of glucose in medium. A dose-dependent on glucose contents for the rescue showing lack of glucose in these seedlings owing to the catalytic mutations. *nin8*+*OsNIN8* without glucose as control. bar = 6 mm. (M) Reducing photosynthetic sugars worsened sugar deficiency. Seedlings of a catalytic mutation line were roots and leaves removed and inoculated in media showed roots could not regrow without glucose but regrew well with addition of glucose. bar = 6 mm. See also Figure S2.

**Figure 5:**
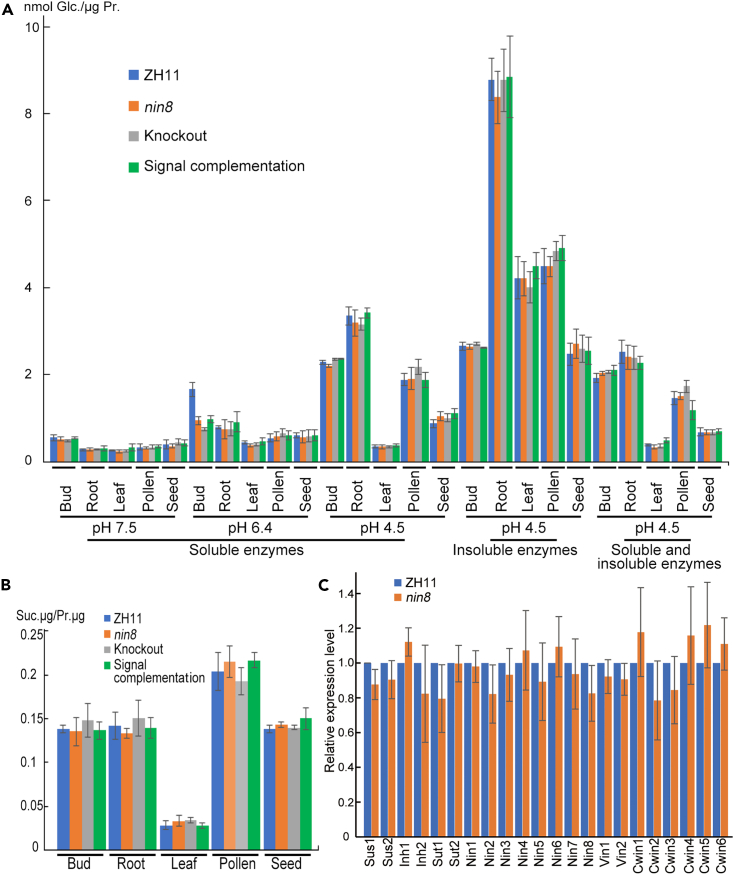
Possible effect of OsNIN8 mutation on genetic compensation for metabolic network of sucrose (A) Activities of mainly relative enzymes for sucrose hydrolysis in different tissues among different lines. Protein samples of ZH11, *nin8*, *OsNIN8* knockout (CRISPR/Cas9) line and signal complementation line (*nin8*+*NIN8D275AE501A*) from bud (DAI 4), root and leaf of seedling (DAI 17), young panicle at pollen filling stage (7 days before heading), seed at filling stage (5 days after flower) were extracted as soluble and insoluble fractions. Soluble fraction was determined for activities of sucrose hydrolysis at pH 7.5 (alkaline invertases) and pH 6.4 (neutral invertases) from cytoplasm, and pH 4.5 (acid invertase) from vacuole; insoluble fraction was determined at pH 4.5 for acid invertases bound to cell wall; while joined soluble with insoluble fractions at pH 4.5 for possible acid invertases regulated by invertase inhibitors if they were soluble proteins. The activity unit was indicated as produced nmol glucose per μg proteins. Data were from 3 independent experiments and represented as mean ± SEM. (B) Activity of sucrose synthases for sucrose synthesis in different tissues among different lines. Soluble extracted proteins from four rice lines were determined for sucrose production from UDP-glucose and fructose. The activity unit was indicated as produced μg sucrose per μg proteins. Data were from 3 independent experiments and represented as mean ± SEM. (C) Comparison of expression levels of main sucrose metabolism relative genes in bud of ZH11 with those of *nin8*. These genes included two sucrose synthases (Sus1: LOC_Os06g09450; Sus2: LOC_03g28330), two invertase inhibitors (Inh1: LOC_Os10g10620; Inh2: LOC_Os04g49720), two sucrose transporters (Sut1: LOC_Os03g07470; Sut2: LOC_Os12g44370), eight neutral/alkaline invertases (Nin1-8, locus numbers saw Figure S6), two vacuolar invertases (Vin1: LOC_Os04g45290; Vin2: LOC_ Os02g01590) and six cell wall invertases (Cwin1: LOC_Os02g33110; Cwin2: LOC_Os04g33740; Cwin3: LOC_ Os04g33720; Cwin4: LOC_Os04g56920; Cwin5: LOC_Os09g08072; Cwin6: LOC_Os08g06210), expression level of ubiquitin-conjugating enzyme (LOC_ Os02g16040) served as internal standard using quantitative PCR. Data were from 3 independent experiments and represented as mean ± SEM. See also Table S8.

**Figure 6:**
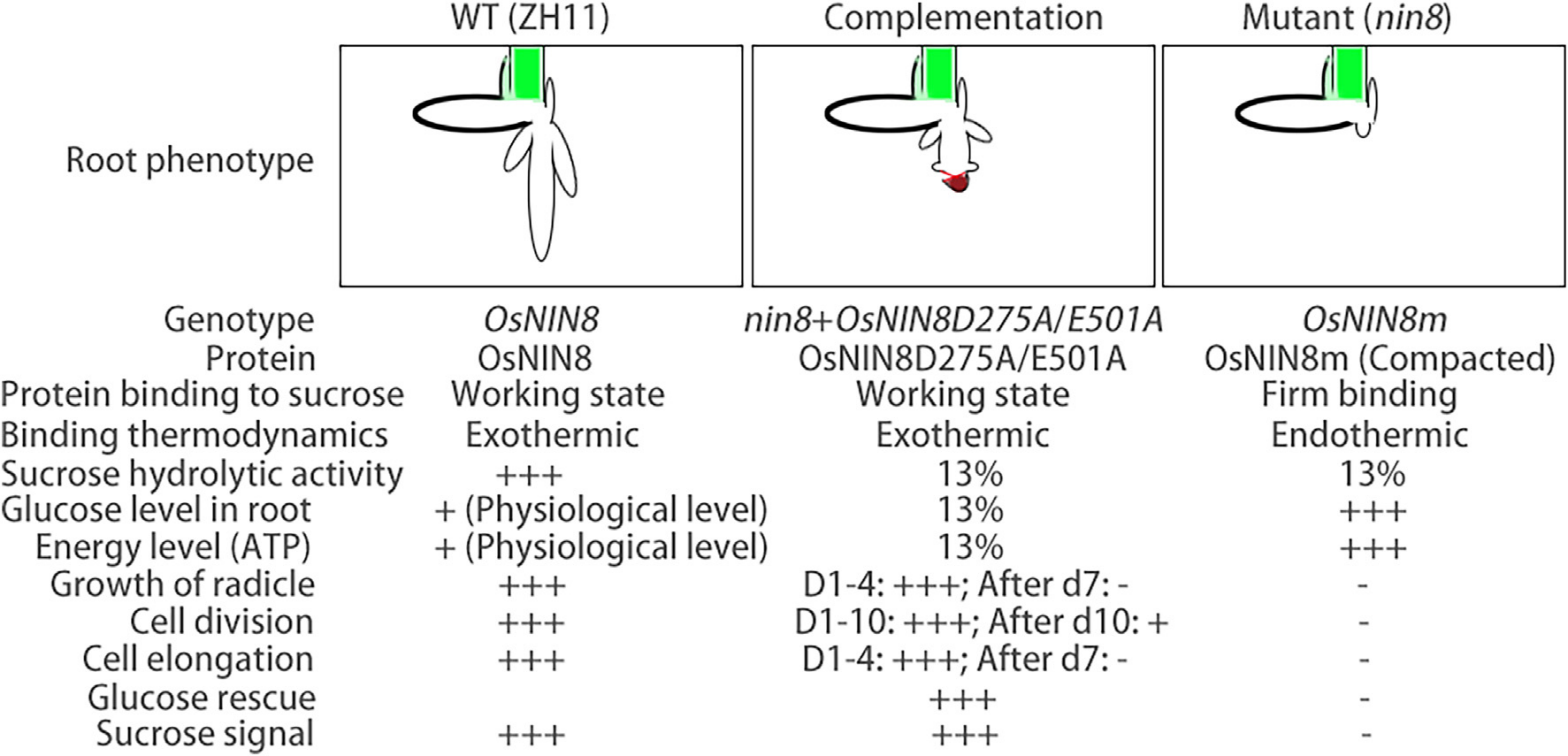
List comparison of sucrose signal generation relating to molecular function of OsNIN8 Showing differences in sucrose binding, binding thermodynamics, levels of nutrition and energy, and cell division among ZH11, *nin8* and complementation of *nin8* with *OsNIN8D275AE501A* line, which was the characterization of sucrose signal.

The mutation G461R was between α-helix_12_ and α-helix_13_ (Figure 1, Figure 2, Figure 3, Figure 4, Figure 5, Figure 6and S9A). OsNIN8 and OsNIN8m were respective modeling and fitted the barrel, Figures S9B and S9C exhibited the openings of inside 6-helixes wall of OsNIN8 and OsNIN8m, respectively. Therefore, a single hydrogen atom side chain of glycine residue at the opening of the barrel of OsNIN8 was displaced by a huge frontier orbital electron cloud of arginine side chain^32^^,^^33^ in OsNIN8m (Figures S9B and S9C). The side chain of arginine is a hydrophobic propyl guanidium group proximally and the strongest positive charge of 20 naturally occurring amino acids distally (Figure S9D). So, the arginine residue blocked across the opening of the barrel with the charge and hydrophobic group in OsNIN8m but the mouth of OsNIN8 was open (Figures S9B–S9C).

To investigate secondary structures of the substitution, proteins with MBP-tag (MBP-OsNIN8 and MBP-OsNIN8m), expressed and purified from *E. coli* (Figures 3A and 3B), were subjected to FTIR analysis. During the amide Ⅰ band, where are sensitive to secondary structural elements of proteins,^34^^,^^35^ MBP-OsNIN8m appeared a random coil at wavenumber of 1645 cm^−1^ but MBP-OsNIN8 had not (Figure 3C). It was mutation induced coils.

Protein folding properties between OsNIN8 and OsNIN8m were compared using nanoDSF assay. Integrated fluorescence of OsNIN8 at both 330 nm and 350 nm were much higher than that of OsNIN8m, particularly at 30°C (biological temperature), namely OsNIN8 were more unfolding and less hydrophobic than OsNIN8m (Figure 3D). Moreover, the melting temperature (Tm) value of OsNIN8 was 56°C, but of OsNIN8m was 60°C (Figure S10). In addition, OsNIN8 had the second Tm at 63.8°C indicating it had second domain (Figure S10), but OsNIN8m had not. Thus, OsNIN8m was compacter than OsNIN8.

Their affinities to sucrose were determined by pulldown assay and bounded sucrose was determined using GC-TOF MS. OsNIN8m bound more sucrose than OsNIN8 did (Figure 3E), namely OsNIN8m bound sucrose firmer and lasted for longer time than OsNIN8 did during the binding for hydrolysis. That was, turnover of sucrose in OsNIN8m was slower than that in OsNIN8.

### OsNIN8 is a neutral invertase with feedback inhibition to sucrose concentration in hydrolysis

Enzymatic activity of OsNIN8 for sucrose hydrolysis was conducted using a microplate method. Firstly, OsNIN8 had the highest activity at an optimum pH of 6.4 (Figure 3F), it belonged to the neutral invertase of pH 6–7.^11^^,^^12^^,^^13^^,^^14^^,^^15^

Microplate and isothermal titration calorimetry (ITC) assays, were used to determine enzymatic activity, it was only OsNIN8 showing a high activity of sucrose hydrolysis and the activity of OsNIN8m was about the background or very low (Figures 3G and 3H).

The Michaelis constant (*Km*) of OsNIN8 for sucrose was 2.718 ± 0.347 mM from microplate method and 1.776 mM from ITC assay (Figures 3I and 3J), which was very low as compared to many known A/N-invertases of 8–30 mM.^11^^,^^12^^,^^13^^,^^36^ Low *Km* indicated that OsNIN8 had a high susceptibility to sucrose concentration.

Interestingly, high sucrose concentration did not maintain OsNIN8 activity, it dropped to and kept a low activity after greater than 20 mM of sucrose (Figure 3J). To confirm this result, the single injection of ITC method was carried out for catalysis assay. Sucrose of 10 mM or 200 mM were injected to OsNIN8. Again, sucrose at valid concentration was converted into products continuously until depletion, but at high concentration sucrose stopped hydrolysis except a high dilution exotherm at the beginning (Figure 3K). So, high concentration of sucrose inhibited activity of OsNIN8.

Similarly, with different concentrations of glucose or fructose in the reaction system as background, high concentrations of glucose or fructose did not inhibit hydrolytic activity of OsNIN8 on sucrose (Figures 3L and 3M).

Consistent with other A/N-invertases,^12^^,^^30^ OsNIN8 specifically hydrolyzed sucrose but not or little on raffinose, maltose and cellobiose (Figure 3N).

### OsNIN8m remains a low sucrose hydrolysis resulting in accumulation of metabolites in *nin8*

To distinguish the short radicle was due to sucrose firm binding only (Figure 3E) or also damage of sucrose hydrolysis in OsNIN8m (Figures 3G and 3H). We mutated OsNIN8 into proteins for loss of sucrose hydrolysis but keeping sucrose binding intact since catalytic residues (D275 and E501 in OsNIN8) and binding residues were independent^30^ inferred from the alignment (Figure S8). D275 and E501 were either respective or both mutated into alanine, namely D275A, E501A and D275AE501A in OsNIN8. These proteins were expressed and purified from *E. coli* (Figure 3B).

Sucrose hydrolysis activities of OsNIN8D275A, OsNIN8E501A, OsNIN8D275AE501A, OsNIN8, OsNIN8m and MBP-tag were determined by microplate and ITC methods (Figures 3G and 3H). OsNIN8 had high activity but MBP-tag, OsNIN8D275A, OsNIN8E501A and OsNIN8D275AE501A showed only background in microplate method. More sensitively, OsNIN8m had a low activity in enthalpy change (heat rate) in ITC assay. Where MBP, OsNIN8D275A, OsNIN8E501A and OsNIN8D275AE501A showed a background of −28.7∽-37.9 μJ, OsNIN8 had its cumulative heat rate of −1257.4 μJ, but OsNIN8m was −199.9 μJ, it was 13% of that of OsNIN8 (Figure 3H). This low sucrose hydrolysis activity of OsNIN8m caused the accumulation of glucose and downstream metabolites in *nin8*. This accumulation disclosed that arrest of cell division was not due to nutritional deficiency.

### Sucrose binding with OsNIN8m is endothermic but with OsNIN8 is exothermic

For thermodynamic analysis of sucrose bindings, an ITC binding equilibrium method was performed by titrating OsNIN8 or OsNIN8m with sucrose. For OsNIN8, exotherm of every titration was due to its sucrose hydrolytic activity and never reaching equilibrium. For OsNIN8m, who had 13% sucrose hydrolysis (Figure 3H) but firm sucrose binding (Figure 3E), its peak-heights were lower than MBP background before titration 10, because its endotherm of sucrose binding was offset by the exotherm of sucrose hydrolysis before reaching equilibrium at titration 10. After then with sucrose accumulating, the binding was greater than the hydrolysis effects, it appeared a net endotherm (Figure 3O). So, OsNIN8m binding to sucrose was endotherm.

Three OsNIN8D275A/E501As, all had not hydrolytic activity on sucrose, showed their bindings to sucrose were exotherm, where the binding sites of proteins were occupied gradually by sucrose with the titration and reached an equilibrium, so the binding pattern exhibited that the exothermic peak-heights were getting lower and lower and then stable (Figure 3P). This pattern indicated the characteristic of interactions of three proteins with sucrose. A reasonable inference of OsNIN8 titrating with sucrose was also exotherm since binding residues of OsNIN8 were the same with those of three OsNIN8D275A/E501As.

### OsNIN8 binding sucrose allows cell division and hydrolysis of sucrose provides nutrition

To character these mutated proteins for their sucrose binding without hydrolytic activity *in vivo*, we transformed three constructs into *nin8* for complementation of *OsNIN8m* with *OsNIN8D275A*, *OsNIN8E501A*, and *OsNIN8D275AE501A*, respectively (Figure 4A).

As compared to complementation line of *nin8* with *OsNIN8*, germination and growth of complementation lines of *nin8* with three *OsNIN8D275A*/*E501A*s showed no difference including roots and above ground parts during early four days (Figure 4B). But, they slowed down elongating after DAI 4 and stopped growing with root tips turning brown after DAI 7 (Figure 4C). At DAI 10, these roots were only 1/3 in length of the control (Figure 4A). After magnifying, it showed ring fold and burst around meristem zone, asymmetric enlargement, crook and lateral root emerging close to basipetal parts of the meristem zone, and browned (Figures 4D and 4E). *nin8* was dwarf all the time (Figures 4A–4C).

These malformed root tips were sectioned and examined. It was found that cells stopped elongating in elongation zones but cell division continued in meristem forming an expanded meristem where it spread tissues around and formed breaches. Where meristematic cells were smaller, more homogeneous than elongated cells, and with heavy nucleus, they piled up and disorderly squeezed forming swellings in root tips (Figures 4F–4H).

We considered that cell division might be a priority to or occur at a lower level of energy for longer time than cell elongation did. So, cell elongation stopped before DAI 4 but cell division continued after DAI 7 in these *nin8*+*OsNIN8D275A*/*E501A* lines. The low nutrition supply was derived from the 13% remnant of sucrose hydrolysis in *nin8*, which accumulated glucose, citric acid and amino acids in *nin8* but could not maintain normal growth in these lines any longer during germination.

To confirm exhaustion of energy in these complementation lines, levels of ATP, ADP, and AMP in roots were measured using UPLC-Q-TOF/MS method. Ratios of AMP/ATP and ADP/ATP of *nin8*+*OsNIN8D275A*/*E501A*s lines were significantly greater than that in control (Figures 4I–4K), indicating lack of energy (rich in AMP or ADP). Interestingly, the ATP in *nin8* was rich, because its cell division was block and the energy was accumulated in cells, it was consistent with accumulations of glucose, citric acid and amino acids in *nin8* (Tables 1 and S2–S7).

To confirm the stops of cell elongation as well as cell division were due to unavailability of glucose. Seeds of *nin8*+*OsNIN8D275AE501A* line were inoculated without or with glucose at different concentrations (Figure 4L). Root length of these shoots were in direct proportion to glucose levels. To minimize photosynthetic sugars, shoots with blades and roots cut off were inoculated without or with glucose. They could not regrow their roots without glucose but regrew well with glucose (Figure 4M), showing depletion of glucose.

Therefore, OsNIN8 and OsNIN8D275A/E501As, which were not compact but exothermic binding with sucrose, allowed cell division regardless of levels of energy and nutrients; OsNIN8m, which was compact and firmer binding with sucrose for endothermic reaction, arrested cell division regardless of supply nutrition or not in rice. Certainly, hydrolysis of sucrose for energy and nutrients made cell division and elongation continuously, though cell division could tolerate lower levels of energy and nutrients than cell elongation.

Similar to the recoveries of radicle growth and seed setting in complementation of *OsNIN8m* with *OsNIN8*, lines of complementation of *OsNIN8m* with *OsNIN8D275A/E501A*s also recovered their radicle growth and seed setting to levels of WT after sugar supply from photosynthesis (Figure S2). So, the manner of OsNIN8 controlling both cell division and sucrose hydrolysis was not only in radicle during germination but also during seed setting.

### Genetic compensation does not appear in the mutation of *OsNIN8*

Recently, a phenomenon of genetic compensation or called transcriptional adaptation was observed in zebrafish and mouse that organisms responded to gene mutations by upregulation of related genes.^37^ Carefully, we had noticed that sucrose, maltose, glucose, fructose, citric acid and amino acids accumulated in sprouts of *nin8*, though these nutrients provided from endosperm always accumulate transiently in these tissues during germination, might partially resulted from genetic compensation.

To survey this compensation, we assayed activities of alkaline invertases, neutral invertases and acid invertases (vacuolar) of soluble fraction proteins, cell wall acid invertases of insoluble proteins, and possible invertase inhibitors regulating acid invertases by combined soluble and insoluble proteins in ZH11, *nin8*, knockout line and signal complementation line (*nin8*+*OsNIN8D347AE501A*) from sprout, root and leaf of seedling, spikelets during pollen filling and grain filling stages (Figure 5A). It indicated that activities of these enzymes in unit proteins did not appear statistically significant difference between the wild-type and gene modified lines except neutral invertases in sprout proteins, where the activity of ZH11 was about twice of that of other lines. This protein preparation contained either OsNIN8 or modified OsNIN8s. The activity level of knockout line might indicate the total activities of other invertases in this non-optimum pH condition.

Similarly, activity of sucrose synthases for direction of sucrose synthesis were assayed in different tissues of four rice lines (Figure 5B). They also did not show significant difference between ZH11 and three gene modified lines.

It was only neutral invertases in sprout showing activities lower in *OsNIN8* modified lines than in ZH11, to survey transcriptional adaptation was involved in this change or not, expression of many genes related to sucrose metabolism were assayed. They included two sucrose synthases, two invertase inhibitors, two sucrose transporters, eight neutral/alkaline invertases, and two vacuolar and six cell wall acid invertases (Figure 5C; Table S8). Expression of these genes in *nin8*, that accumulated sugars in sprout (Tables 1 and S2–S7), were not upregulated significantly as compared to their levels in ZH11.

Altogether, these data indicated that genetic compensation was not involved in this mutation.

## Discussion

Carbohydrate partitioning from source tissues to sink tissues is a spatial and temporal dynamics of the underlying physiological processes during the life cycle in rice.^38^^,^^39^ Many signal pathways including auxin and stress tolerance are involved in regulation of this partitioning.^40^^,^^41^^,^^42^ So, it is very complex for sugar signaling in these tissues.

However, it is a one-way catabolic flow of storage starch in the seed and then anabolic flows of metabolites, such as organic acids, amino acids and energy for cell division in radicle and plantule during germination. And sucrose, which is the form of long distant transport carbohydrate, links the two processes in starchy seeds. Thus, it is a relatively simple model for investigating sucrose catabolism to maintain cell division during germination in rice.

### Identification of subinitial in cell lineages facilitates observation of cell division for root growth

Functional organization of the structure of root apex is confused so far and it is difficult to observe times of cell division for root elongation. Many attempts have been made to segment the meristem into layers, but the results were controversial. In addition, a QC in the center puzzled the delimitation of number and position of initials in the meristem.^43^

Here, roots of *nin8* was short but not small. The enlargement of root apex came from cell division of the initial for number of cell lineages in transection and the elongation of root tip came from cell division of subinitials in axial cell lineages. After then, cellular differentiation of a cell lineage was decided by its position. Because patterning of a cell lineage in meristem followed the patterned cell lineage axially, which it was end to end connection with basipetally.^44^ Therefore, division ratio of initial to subinitials determined diameter and length of root. Propulsive force from cell growth shaped the dome of root apex.

The growth of a root from an initial could be expressed as a mathematical formula:

R=abcIS

Here a, b and c are the length, width and height of a cell; and I and S are division times of the initial and subinitial in axial cell lineages, respectively. So, I and S are only variables of function R; a, b and c are constants. Root length = aS; Root diameter = bcI. Value ranges of I and S determine root length and diameter concurrently.

Subinitials in the center of cell lineages and cells with EdU incorporation getting longer with the treatment time proved this fashion of cell division. Similar distribution of labeled cells was observed early in root exposed to tritiated thymidine showing the QC and occasional labeled cells present within the QC.^45^ With this pattern, root was constructed and organized by all kind of cell lineages one by one.

Interestingly, if more seeds were inoculated in a small test tube, roots would disperse each other and grow toward the tube wall in larger angles, where they curled into helical growth (Figure S11). Therefore, we believed that periclinal division of initial producing subinitials to build the root body with cell lineages must follow a direction around the initial, so it produced a partial propulsive force continuously around the root during root elongation. With this order, it would be good for stacking cell lineages in the root. And the root tips were easy to curl when they were stopped by a flat surface. The alternative possibility was a positive response, that directional division of initial and/or division times of subinitial in one direction changed when root tips met an obstacle and tried to find ways out. The touch continued and the orientation continued forming a helix. Interestingly, lengthening of root length was contributed mainly from cell elongation but not cell division, so the tips of curly root always showed straight (Figure S11).

These findings were obtained from observation of *nin8* for understanding of root apex organization, in turn, it made us clear that mutation of sucrose signal in *nin8* arrested division times of subinitials for elongation but not that of the initial for root enlargement.

### Only OsNIN8 hydrolyzes sucrose to supply glucose and energy for germination

OsNIN8 is a cytosolic invertase, and sucrose moves symplastically to cytoplasm of dividing cells in root tips, where sucrose is degraded to produce energy and materials for germination. Sucrose could be degraded either by invertases or sucrose synthases. All isoforms of sucrose synthase did not restrict but cytosolic neutral invertases restricted growth of root during germination in *A*. *thaliana*.^20^ Here, rice lines complementation of OsNIN8m with OsNIN8D275A/E501As, which lost the function of sucrose hydrolysis, were deficient in glucose and energy to stop root elongation, this deficiency could be rescued by adding glucose into the media during culture. Additionally, knockout of the rest of OsNINs (OsNIN1-7) individually did not appear similar phenotypes of root arrest from nutrition exhaustion. Therefore, it was only OsNIN8 but not sucrose synthase and other invertases contributed to sucrose catabolism in root tips during germination in rice.

Interestingly, there were enzymic activities of acid, neutral and alkaline invertases, and sucrose synthases in rice sprout, other enzymes could not replace OsNIN8 to provide energy for cell division during germination, which might be due to the compartmentalization.

### Signal effect can be distinguished from nutrition effect of sucrose

Sucrose as a signaling molecule in plants has been concerned for long time, but generally accepted until from some clues of sucrose metabolism.^10^^,^^46^ Recently, it was suggested that sucrose is a preferred signaling molecule for syntheses of starch, anthocyanin, ribosome and some storage proteins, sucrose transport, cell division and floral induction in plants, but little is known about the molecular mechanisms underlying such as direct sucrose sensor.^47^^,^^48^ The challenge is distinguishing the signal effect from the nutrition effect of sucrose.

Here, within catabolic flow of sucrose for cell division during germination, OsNIN8 controlled not only levels of energy and glucose for cell elongation as well as cell division from nutrition effect but also effect of cell division, which was require of activation cell signal system. Importantly, two effects were independent each other, *nin8* (OsNIN8m) lost function of cell division but its nutrient accumulation remained from the residual 13% sucrose hydrolysis activity, while lines of complementation of OsNIN8m with OsNIN8D275A/E501As lost nutrient supply except the residual 13% activity from OsNIN8m for DAI 1–4, which maintained cell division and elongation at early stage until used out of nutrition, but its signal effect on cell division remained intact continuously. Nutrition effect could be surveyed by determining levels of metabolites such as glucose and ATP or adding glucose in the medium during culture, and signal effect could be observed by the length of cell lineages or size of meristem. Derivation as well as manifestation of two effects were clear and different in these rice lines. They could not be distinguished from each other in WT.

### Sucrose signal for cell division is not be triggered in *nin8*

In WT, OsNIN8 was intact, OsNIN8 binding to sucrose and hydrolysis sucrose were naturally occurred, levels of glucose and energy were as physiological, and cell division and elongation were as normal for growth, its sucrose signal was working for cell division (Figure 6).

In *nin8*, OsNIN8m was hydrophobically compacted and firm binding to sucrose, levels of glucose and energy were high owing to its low activity of sucrose hydrolysis, notwithstanding, cell division was arrested, resulting from the sucrose signal was not triggered for cell division in *nin8* (Figure 6).

Interestingly, phenotypes of stunted radicle during germination and low seed setting in lines of *OsNIN8* knockout were similar to *nin8*, it indicated that the compacted OsNIN8m completely blocked the sucrose signal for radicle growth and seed setting just like absence of the gene.

In lines of complementation of OsNIN8m with OsNIN8D275A/E501As, these mutated OsNIN8s could normally bind to sucrose but could not hydrolyze sucrose to glucose, so they were deficient in glucose and energy, but cell division was normal occurrence before exhaustion of nutrients and energy, therefore its sucrose signal for cell division was working (Figure 6). Therefore, OsNIN8 sensed sucrose as signal in this process in rice.

### Signal initiating indicates an association of sucrose with OsNIN8 dissipating energetic strain

In theory, signal sensing is a process of interaction between surfaces of ligand (e.g., sucrose) and receptor (e.g., OsNIN8). A ligand binding to the receptor lead to dissipate the energetic strain at the allosteric perturbation site initiating signal cooperatively for positive or negative, up- or down-regulating function.^49^ The driving basis underlying protein-ligand recognition and binding is its thermodynamic properties.^50^ It is hydrogen bond, hydrophobic association, van der Waals, ionic and protonation that contributes to thermodynamics of protein-ligand and protein-protein associations.^51^^,^^52^ Mutation at glycine^461^ into arginine of OsNIN8m altered its surface properties, which alter the association between OsNIN8m and sucrose into a firmer binding, and stopped the signal for cell division.

OsNIN8m induced random coil at 1645 cm^−1^, lost its second domain, and had higher Tm and was compacter than OnNIN8, led to a tighter binding to sucrose.

Thermodynamically, OsNIN8m interacting with sucrose was endothermic, which arrested cell division; OsNIN8D275A/E501As or OsNIN8 interacting with sucrose were exothermic, which allowed cell division. Because exothermic is energetically favorable for formations of interactions between atoms^50^ it might dissipate the energetic strain of sucrose-OsNIN8 interaction in a loose state. Therefore, exothermic association of OsNIN8 with sucrose allowed initiation of sucrose signal but endothermic association of OsNIN8m with sucrose extinguished the signal.

### Limitations of the study

Physiological processes of reproductive development involved a transition from vegetative to reproductive phase,^53^ panicle differentiation including differentiations and growths of branches, glume, male and female organs, microspore, and megaspore,^54^ fertilization and grain filling. Interestingly, sucrose in the medium affected floral transition in *Arabidopsis*.^55^ Again, sucrose supported pollen germination but glucose inhibited pollen germination *in vitro*, which involved an HXK signaling pathway in *Arabidopsis*.^56^ It suggested that sugar signaling involved in reproductive development in *Arabidopsis*.

In the current work, arrest of sucrose signal (OsNIN8m) impaired pollen development and seed setting in *nin8* but OsNIN8D275AE501A (sucrose signal was working) recovered seed setting in *nin8*+ *OsNIN8D275AE501A* lines. Additionally, developments of panicle, florets and pollen were susceptible to low temperature in *nin8* but were not in WT. The molecular mechanism underlying these phenotypes would be informative.

## STAR★Methods

### Key resources table

**Table:** 

REAGENT or RESOURCE	SOURCE	IDENTIFIER
**Antibodies**		

Anti-MBP (Maltose Binding Protein) mAb	MBL	Cat# M091-3; RRID: AB_2770406
Goat anti-mouse IgG-HRP secondary antibody	Santa Cruz	Cat# SC-2031; RRID: AB_2716555

**Bacterial and virus strains**		

*E.coli* DH5α competent cell	TIANGEN	Cat# CB101
BL21(DE3) chemically competent cell	TRANSGEN	Cat# CD601

**Chemicals, peptides, and recombinant proteins**		

Tryptone	OXOID	Cat# LP0042B
Yeast extract powder	OXOID	Cat# LP0021B
D-Glucose, anhydrous	Mei5bio	Cat# G47857
Sucrose	MP Biomedicals	Cat# 04821721
D-Mannitol	Sigma-Aldrich	Cat# M9647
D-Raffinose	Lablead	Cat# J392
Cellobiose	Macklin	Cat# D805315
Amylose Resin	Biolads	Cat# E8021
Amylose magnetic beads	Biolands	Cat# E8035
Click-iT EdU imaging kits	Invitrogen	Cat# C10337
Glucose detection kit	Abcam	Cat# ab102517
Glucose detection kit	Abcam	Cat# ab65333
Sucrose assay kit	OKA, Beijing, China	Cat# D17209
RNeasy plant mini kit	Qiagen	Cat# 74903
SuperScript Ⅲ first-strand synthesis system	Invitrogen	Cat# 18080-051
Power SYBR™ Green PCR Master Mix	Applied Biosystems	Cat# 4367659
UDP-glucose	Abcam	Cat# ab120384
Sucrose standard	Sigma-Aldrich	Cat# V900116
Glucose standard	Sigma-Aldrich	Cat# G8270
Fructose standard	Sigma-Aldrich	Cat# PHR1002
Maltose standard	Fluka	Cat# 8033
Citric acid standard	Sigma-Aldrich	Cat# 27102
Amino acids standard	FLUKA	Cat# AAS18
ATP standard	Sigma-Aldrich	Cat# A2383
ADP standard	Sigma-Aldrich	Cat# A2754
AMP standard	Sigma-Aldrich	Cat# A1752

**Experimental models: Organisms/strains**		

*nin8* rice mutant	This paper	*nin8*
*nin8*+*OsNIN8* complementation line	This paper	*nin8*+*OsNIN8*
*nin8*+*OsNIN8D275A* complementation line	This paper	*nin8*+*OsNIN8D275A*
*nin8*+*OsNIN8E501A* complementation line	This paper	*nin8*+*OsNIN8E501A*
*nin8*+*OsNIN8D275AE501A* complementation line	This paper	*nin8*+*OsNIN8D275AE501A*
*OsNIN8* CRISPR/Cas 9 knockout line 1	This paper	*OsNIN8 CRI1*
*OsNIN8* CRISPR/Cas 9 knockout line 2	This paper	*OsNIN8 CRI2*
*OsNIN8* RNAi knockdown line	This paper	*OsNIN8 RNAi*
*OsNIN8* overexpression line	This paper	*OsNIN8 OE*

**Oligonucleotides**		

Marker RM6509F	Tsingke	GGTGTTTTGTGGTGTTGTGC
Marker RM6509R	Tsingke	CTCGAACTGCGAGTAGGACC
Marker RM2634F	Tsingke	GATTGAAAATTAGAGTTTGCAC
Marker RM2634R	Tsingke	TGCCGAGATTTAGTCAACTA
Marker 398F	Tsingke	GGAAGAGGAAGGTCGGTGATGG
Marker 398R	Tsingke	CGGCGAACATGTCGTTCATCC
Marker 402F	Tsingke	GGGCATGCTCTACCACTCTTACC
Marker 402R	Tsingke	CTCACCAAAGATTCGGTATGTGC
Marker 403F	Tsingke	GTGCTCCTCTCCGTGCTATGC
Marker 403R	Tsingke	TACGTAATGCGGTGCTCCTTGC
Marker 404F	Tsingke	CATAGGCACACCCACTCG
Marker 404R	Tsingke	AATTCAACGGAACAGAACTGTC
Marker 242F	Tsingke	ATTCTTCCTCGTGAGGTGA
Marker 242R	Tsingke	CATGAGCAATAGTACAGGACAAGG
Marker 252F	Tsingke	ATTAACAATGGACCCACAAC
Marker 252R	Tsingke	CAAGCTGATGTCCCTGTC
Marker 262F	Tsingke	CATTCCGTCTCGGCTCAACT
Marker 262R	Tsingke	CAGAGCAAGGTGGCTTGC
Marker 666F	Tsingke	GAAGGTGGAAACTGTTGTTCAGG
Marker 666R	Tsingke	GACAGTTTAGCCTGTACTCCATCC
Marker RM1303F	Tsingke	CTGATCTTGGTGAGCGAGTG
Marker RM1303R	Tsingke	TACGGATCAGCACTCAGCAC
Marker RM3688F	Tsingke	GTTGAATCAAGCTGTGCAGC
Marker RM3688R	Tsingke	AGCTAGGCAAAGCATGCATG
UBIF	Tsingke	ACTCTCAATTTGCTCGCTGTTG
UBIR	Tsingke	AATCTCTGGAACCAATGGATCATC
SUS1F	Tsingke	AGCCATGACATGTGGTTTGC
SUS1R	Tsingke	ATCTCAGCAGGGCCACCAT
SUS2F	Tsingke	GAAACCCGCCGCTACCTT
SUS2R	Tsingke	GCTAGCCATGGTGCGGTACT
INH1F	Tsingke	CGTCTGCTACGACTCCC
INH1R	Tsingke	CGTGGCTGGTCTGGAAC
INH2F	Tsingke	CCGCTGGTTGCGGAGTAC
INH2R	Tsingke	CTATCAATGCGAGAGCAATGGA
SUT1F	Tsingke	GGTGACCCAAAGGGAACTGAT
SUT1R	Tsingke	TGCCCTGACACCCTGGTT
SUT2F	Tsingke	CACCGAGAATGACCCAAAGAG
SUT2R	Tsingke	AGGGCCATGAACAATGAGAAGT
NIN1F	Tsingke	CCATGGGCATCCTCTTGAGA
NIN1R	Tsingke	GGGCACAACGCAAAGCA
NIN2F	Tsingke	GGCTGTGCCTCTTGATGACA
NIN2R	Tsingke	TCTCCAAAGTCAGGGTCCAAA
NIN3F	Tsingke	AGTCCGGTCGGGACGATT
NIN3R	Tsingke	ATAATTCATCGGATTGGCATCAT
NIN4F	Tsingke	AAGGGTTGATGCCAGCAAGT
NIN4R	Tsingke	CATCATCGTCCCCGTCAAGT
NIN5F	Tsingke	TTCTGCCTGGGCAACTTCA
NIN5R	Tsingke	GCCTGTTCTCCGGTTGTCA
NIN6F	Tsingke	TTTGCCCTTGGAAATTGCA
NIN6R	Tsingke	TGCCACTGATTGCTCTGGAGTA
NIN7F	Tsingke	TGCCGCTCAAGATCAGCTT
NIN7R	Tsingke	GCACCCGGTGACGAACTC
NIN8F	Tsingke	CGCCGGAATGCCAGAAG
NIN8R	Tsingke	ATCAAACCCCTCAGACAAGCA
VIN1F	Tsingke	CGTCGGCAAAGCAACACA
VIN1R	Tsingke	CCGAAGTGTCCACCTCGAA
VIN2F	Tsingke	GCGCACCGGATTCCATT
VIN2R	Tsingke	CGTTGGGATCGTTCATCCA
CWIN1F	Tsingke	ATTCCGCGATCCGACAAC
CWIN1R	Tsingke	AACGAGCATCCGCCAATG
CWIN2F	Tsingke	CGTTTGCAGGGTTCGTTGA
CWIN2R	Tsingke	CGATCAGGCTCCTCAGAGATATC
CWIN3F	Tsingke	CATGTGCAACGACCCTACCA
CWIN3R	Tsingke	GCGAAGGTCGGCCTGTAGA
CWIN4F	Tsingke	CGGGTACACGGTCCTCATGT
CWIN4R	Tsingke	GTACACCCCTGCTCTTGAAGTTG
CWIN5F	Tsingke	CGGCGTGCAGACATTCC
CWIN5R	Tsingke	CAGCTGCTTGCCGTCCTT
CWIN6F	Tsingke	GCCGCCCATGATGAACTG
CWIN6R	Tsingke	GCGGAGGCAATGCACAAT

**Recombinant DNA**		

Plasmid: pETMALc-H::*OsNIN8*	This paper	*MBP-OsNIN8*
Plasmid: pETMALc-H::*OsNIN8m*	This paper	*MBP-OsNIN8m*
Plasmid: pETMALc-H::*OsNIN8D275A*	This paper	*MBP-OsNIN8D275A*
Plasmid: pETMALc-H::*OsNIN8E501A*	This paper	*MBP-OsNIN8E501A*
Plasmid: pETMALc-H::*OsNIN8D275AE501A*	This paper	*MBP-OsNIN8D275AE501A*
Plasmid: pCAMBIA 1390::*OsNIN8*	This paper	*OsNIN8* comp.
Plasmid: pCAMBIA 1390::*OsNIN8D275A*	This paper	*OsNIN8D275A* comp.
Plasmid: pCAMBIA 1390::*OsNIN8E501A*	This paper	*OsNIN8E501A* comp.
Plasmid: pCAMBIA 1390::*OsNIN8D275AE501A*	This paper	*OsNIN8D275AE501A* comp.
Plasmid: pTCK303::*OsNIN8*^*789-*^*^1050^*^*bp*^ RNAi	This paper	*OsNIN8* RNAi
Plasmid: pCAMBIA 1391::*ProUBI*-*OsNIN8*	This paper	*OsNIN8* OE

**Software and algorithms**		

AlphaFold2	DeepMind	AlphaFold2
SWISS-MODEL	SIB and Biozentrum	SWISS-MODEL
NanoAnalyze software v3.7.5	TA Instruments	v3.7.5

**Other**		

Affinity ITC	TA Instruments	ITC LV
Microplate reader	TECAN	Infinite M200PRO

### Resource availability

#### Lead contact

Requests for resources and reagents should be directed to the lead contact, Zizhang Wang (zizhangw@ibcas.ac.cn).

#### Materials availability

This study did not generate new unique reagents.

#### Data and code availability

• Data reported in this publication will be shared upon request from the lead contact.
• This study did not generate unique code.
• Any additional information required to reanalyze the data reported in this paper is available from the lead contact upon request.

### Experimental model and study participant details

#### Rice materials and growth conditions

For selection of phenotypes of rice seedling’s root, we developed a consistent culturing condition based on Yoshida semisolid medium^57^ in 2.5 × 25 cm test tube at 24°C (imitating underground temperature) with 12h–12h light-dark cycle at about 50 μmol/m^2^.s of light intensity in a plant culture room.

Rice unshell seeds were sterilized with 70% alcohol briefly and sodium hypochlorite (1% effective chlorine) for 30 min and washed with sterile water for three times, and inoculated. Or rice seeds were placed in a cut-off bottom 96-well plate separately and kept seeds semi- immersion in water. Seedlings were transplanted to experimental field after acclimatization for a few days.

A population (M1 generation) of sodium azide (0.2 M) treatment of ZH11, a japonica rice, was kindly provided by Prof. Qi Xiaoquan’s lab of Institute of botany, Chinese academy of sciences. And *nin8* mutant was obtained after screening. *nin8* was hybridized to NJ6, an indica rice, to establish F2 segregant population. Based on segregants with *nin8* root phenotype the map-based cloning strategy was carried out to clone the gene.

A 6.6 kb genomic fragment of *OsNIN8* was cloned from ZH11 and subcloned into pCAMBIA 1390 vector and then transformed into *nin8* using *Agrobacterium*-mediated method to obtain complementation lines. Both +939A and +2887A of *OsNIN8* genomic sequence were mutated into cytosine (C) mediated by PCR primers to obtain D275A and E501A amino acids mutation on catalytic residues for sucrose hydrolysis of the enzyme. Three constructs including D275A, E501A and both D275AE501A mutations were transformed into *nin8* for complementation.

A CRISPR/Cas9 approach to knock out two targets on *OsNIN8* of ZH11 were introduced by Biogle company (Hangzhou, China). *Cas9* gene in knock out lines were eliminated by crossing these plants with ZH11. Genotypes of segregant were confirmed by PCR and sequencing. Similarly, *OsNIN1*-*7* in ZH11 were performed knockout.

A 262 bp cDNA sequence (789-1050 bp on *OsNIN8*) was forward and reverse inserted into pTCK303^58^ for *OsNIN8* RNAi construct. The *OsNIN8* CDS driving by UBI promotor and inserted into pCAMBIA 1391 vector was constructed for over expression. These constructs were transformed into ZH11.

Sampling of tissues for enzymatic activity and quantitative PCR. Seeds were inoculated on wet filter paper for 4 days in dark at 24°C, sprouts were removed of endosperm and collected as sprout samples. Seeds were semi-immersion cultured in water for 17 days, and roots and leaves were collected as seedling samples. Rice lines of different genotypes were planted in the same plot in the field, middle segment of young panicles at 7 days before heading were collected as sample of pollen filling tissue; seeds at 5 days after flower were collected as seed filling samples.

### Method details

#### Semi-thin section preparation and microscopic observation

Root tips of rice at 10 days old (DAI 10) were fixed with FAA solution, dehydrated with ethanol, displaced with acetone, permeated with Spurr resin and embedded. Section was performed using Leica RM2255 microtome in 1 μm and stained by toluidine blue O. Microscopic examination was using Zeiss Axio Imager A1.

#### DNA synthesis measured by EdU incorporation

EdU assay was performed by using of Click-iT EdU imaging kits (Invitrogen, C10337). Roots of 10-day rice shoot were labeled with 20 μM EdU for 30 min, 3 h or 10 h. Root tips were fixed in 4% paraformaldehyde with 0.1% Triton x-100 and 0.1% Tween-20 for 1 h (vacuumizing). The Click-iT reaction and DAPI counterstain were performed for 1 h each following the instruction of the kit. A clearing with 6 M urea containing 30% (v/v) glycerol and 0.1% (v/v) Triton-100^59^ was performed with gentle rotation at 4°C for 8 days. Imaging was visualized using Leica TCS SP5 confocal laser scanning microscopy with 495 nm excitation.

#### Determination of carbohydrates, citric acid and free amino acids

Determination of sucrose, maltose, glucose, fructose, citric acid and free amino acids were performed as described previously.^60^

For carbohydrates and citric acid, samples (about 100 mg) added 100 μg ribitol as an internal standard and homogenized in 0.75 mL 80% MeOH with the FastPrep-24 (Irvine, California, USA) for 1 min, and supernatants were collected after centrifugation at 18 000 × g for 10 min. The remaining residue was treated twice as described. The pooled supernatant was evaporated using a concentrator (Speedvac System, NC, USA) to dryness and redissolved in 500 μL of ddH2O, then extracted with chloroform (1:1). A volume of 50 μL of aqueous phase and dilutions of the standards (Sigma) were dried and derivatized with 50 μL (20 mg/mL) methoxylamine hydrochloride (Sigma) in anhydrous pyridine (Sigma) at 30°C for 1.5 h and then 80 μL of N-methyl-N-(trimethylsilyl)-trifluoroacetamide (Sigma) was added at 37°C for 30 min. The derivatives were analyzed by GC-TOF-MS (LECO Corp., USA) within 24 h.

For free amino acids, norvaline of 15 μg was added to 100 mg of sample powder as an internal standard, then samples were homogenized with 0.75 mL of 70% MeOH (v/v) with FastPrep-24 for 1 min, sonicated for 5 min, and centrifugation; the supernatant was collected. The extraction was repeated for three times. and supernatants were pooled and adjusted to pH 2.0 with 0.5 N HCl. The pooled supernatant was extracted with a mixture of light petroleum/diethyl ether (1:1, v/v) three times, then the aqueous phase and dilutions of the standard solution (Sigma, St. Louis, MO, USA) were evaporated to dryness. Amounts of 20 μL of dimethylformamide and 80 μL of N-tertbutyldimethylsilyl-N-methyltrifluoroacetamide (both Sigma) were added to the residue for derivatization at 70°C for 25 min and analyzed by GC-QqQ-MS/MS (Agilent, Palo Alto, CA, USA) within the next 24 h.

#### Protein purification and characters

*OsNIN8*, *OsNIN8m*, *OsNIN8D275A*, *OsNIN8E501A*, and *OsNIN8D275AE501A* were cloned in-frame into pETMALc-H^61^ to generate MBP fusion proteins. Expression of these proteins were using *E.coli* BL21 (DE3) (Biolabs) in LB medium with 0.1 mM IPTG at 25°C for 4 h. Proteins were purified using Amylose Resin (Biolads, E8021) and dialysed using 12–14 kD dialysis membrane (Spec tra/Por) in 50 mM potassium phosphate buffer (pH6.4) for 8 h and renewal buffer for another 4 h, stirring at 4°C. Protein concentrations were determined by Braford reagent (Sangon, Shanghai, China).

#### Fourier transform infrared (FTIR) spectroscopy assay

FTIR spectroscopy was performed as described.^62^^,^^63^ Proteins for concentrating and reducing salt ions were performed using Microcon centrifugal filter unit (Millipore) and washed with 5 mM potassium phosphate buffer (pH 6.4) for four times. The buffer was replaced with D_2_O buffer containing 50 mM K_2_DPO_4_/KD_2_PO_4_ (pD 6.4) for five times. Concentration of these proteins were adjusted to 0.1 mM each. FTIR spectra were collected on a spectrometer (ABB-BOMEM, Bureau, Canada) equipped with a liquid nitrogen cooled broad band mercury-cadmium-telluride detector. A two-compartment CaF2 sample cell with a 56-mm thick Teflon spacer was used for the protein solution and reference D_2_O or proteins, respectively. An average of 50 scans was taken for each spectrum.

#### Nano differential scanning fluorimetry (nanoDSF) assay

MBP-OsNIN8 and MBP-OsNIN8m of 0.1 mM in 50 mM potassium phosphate buffer (pH 6.4) were loaded into the capillaries in duplicate each. Fluorescence at 330 nm and 350 nm were recorded by Prometheus NT.48 (NanoTemper, München, Germany) ranged from 30°C to 95°C. The ratio of the two wavelengths was plotted against the temperature. The first derivative was deduced to determine the Tm of the protein by the software.

#### Pulldown assay of sucrose binding to OsNIN8 and OsNIN8m

Binding of 0.5 mL 0.1 mM MBP, MBP-OsNIN8 and MBP-OsNIN8m to 150 μL amylose magnetic beads (Biolands, E8035) each and added 100 μL 1 M sucrose to the beads mixing at 25°C for 5 min. These beads were washed with 1 mL 4°C, 50 mM potassium phosphate buffer (pH 6.4) three times on ice, added 100 μL buffer and transferred into boiling water for 5 min. The samples were subjected to GC-TOF-MS for determination of sucrose, glucose and fructose as described previously.^60^ The experiment was repeated 3 times independently.

#### Microplate assay of invertase activity

Determination of glucose for sucrose hydrolysis of OsNIN8 involved was use of a glucose detection kit (Abcam, ab102517) using a microplate reader (TECAN infinite M200PRO) at 450 nm. The reaction was performed using 5 μL 0.1 mM enzyme and 5 μL 100 mM sucrose in 50 mM potassium phosphate buffer (pH 6.4) with a total of 100 μL following the instruction of the kit. MBP was as the control. Experiments were repeated 3 times independently.

Reactions were carried out in 50 mM potassium phosphate buffer with pH 5.4, 5.6, 5.8, 6.0, 6.2, 6.4, 6.6, 6.8, 7.0, 7.2, 7.4, 7.6, 7.8 and 8.0 to determine optimum pH of OsNIN8.

Reactions were carried out in 50 mM potassium phosphate buffer (pH 6.4) with sucrose concentration of 0, 0.25, 0.5, 1, 2, 4, 5, 10, 20, 40, 100 and 200 mM for enzyme kinetics of OsNIN8. The Michaelis constant (*Km*) of the reaction was generated by fitting to the Michaelis-Menten equation using Origin 93 program (Northampton, MA, USA).

Reactions were carried out with addition fructose or glucose to final 50, 100 and 200 mM for fructose or glucose inhibitions, respectively, when parallel reactions except for the enzyme were boiled to inactivate as references. For glucose inhibition, glucose content of reactions with active enzyme minus those with inactive enzyme were net products of the reaction.

Reactions were carried out by displacing sucrose with raffinose, maltose and cellobiose for substrate specificity.

#### Isothermal titration calorimetry (ITC) assays of sucrose binding to and kinetics of OsNIN8s

ITC assay was performed on Affinity ITC LV (TA Instruments, New Castle, DE, USA) with NanoAnalyze software v3.7.5 (TA Instruments). Protein concentrations in the sample cell were 0.1 mM, sucrose concentration in the syringe were 10 mM, temperature was 25°C. The single injection experiment: 10 μL sucrose was injected to enzyme samples, and reaction for 1 h for kinetics assay. Incremental titration experiment: injection volume was 2.5 μL with interval of 200 s for 20 injections to inject sucrose to proteins for assay of sucrose binding to proteins.

#### ATP, ADP and AMP determination

Roots (0.5 g) of seedling at 17 DAI were ground in liquid nitrogen and extracted with 0.5 mL cool 10 mM ammonium formate containing 0.1% formic acid (v/v). The supernatant of centrifugation (18 000xg, 20 min at 4°C) was collected for analysis using Agilent UPLC-Q-TOF/MS with SB-C18 RRHD column (2.1 × 100 mm 1.8 μm). Column temperature was 35°C. Injection volume was 5 μL. Mobile phase flow was 0.35 mL/min. Mobile phase buffer A was extraction buffer. Mobile phase buffer B was methanol containing 0.1% formic acid (v/v). Gradient of buffer B was 0 min 1%, 0–2 min 1%, 2–3 min 1%–20%. MS conditions: (-) ESI, gas temperature 325°C, drying gas 11 L/min, nebulizer 40 psi, sheath gas temperature 325°C, sheath gas flow 10L/min, capillary voltage 3500 v, fragmentor 125 v.

#### Extraction and fraction of enzyme proteins from tissues, and determination of enzyme activities

Tissue samples (0.10 g) were ground in liquid nitrogen and the powder was resuspended with 1 mL extraction buffer (50 mM HEPES-KOH, pH 7.5, 1 mM EDTA, 3 mM DTT, 3 mM MgCl_2_). The supernatant after centrifugation (14,000xg for 10 min at 4°C) was kept at −80°C as soluble enzyme proteins. The pellet was resuspended in 1 mL extraction buffer and centrifugated to wash for three times at 4°C and finally resuspended in 1 mL of this buffer as insoluble enzyme proteins following a previous description.^64^ Protein contents were determined using Bradford method.

The activities of enzyme proteins for hydrolysis of sucrose were conducted in 100 μL 80 mM buffers (sodium acetate pH 4.5 for acid invertases, sodium phosphate pH 6.4 and pH 7.5 for neutral invertases and alkaline invertases, respectively) contained 100 mM sucrose and 0.5 μg enzyme proteins for 30 min at 37°C. The reaction was terminated at 98°C for 10 min, and 5 μL and 4 μL of 1 M Tris-HCl (pH 10) were added to pH 4.5 and pH 6.4 reactions for neutralization, respectively. Detection of produced glucose using an Abcam kit (ab65333) and the microplate reader at 570 nm was performed to assay activity of enzymes.

The activity of sucrose synthase (direction of sucrose synthesis) was followed a previous description.^65^ The reaction was carried out in a volume of 200 μL containing 55 mM HEPES-NaOH (pH 7.4), 16 mM MgCl_2_, 8 mM fructose, 8 mM UDP-glucose and 5 μg soluble enzyme protein for 20 min at 30°C, and stopped at 98°C for 10 min. The sucrose synthesized was measured using a sucrose assay kit (OKA, Beijing, China. D17209) following the instruction.

Experiments were repeated 3 times independently.

#### Quantitative PCR

RNA isolation was using RNeasy plant mini kit (Qiagen, 74903). cDNA synthesis was using SuperScript Ⅲ first-strand synthsis system for RT-PCR (Invitrogen, 18080-051). Real time quantitative PCR was performed using StepOnePlus system (Applied Biosystems), PCR primers were designed by Primer Express v3.0 tool of the system. The assay was repeated for three times.

### Quantification and statistical analysis

Quantitative ion of mass spectra for ribitol (internal standard, IS), citric acid, fructose, glucose, sucrose and maltose were at 217, 273, 217, 319, 361 and 361 m/z according to fragments of standards, respectively. Peak areas were collected for quantification using LECO ChromaTOF v3.32 (Leco Co., CA, USA).^60^ Quantification of free amino acids was operated in electron ionization in multiple reaction monitoring mode using the Agilant MassHunter workstation (Agilent, Version B.05.00). Mass spectra of precursor ion (m/z) and quantitative product ion (m/z, in bracket) of amino acids were as follows: alanine 260 (232), glycine 16 (218), valine 288 (260), norvaline (IS) 288 (260), leucine 302 (274), isoleucine 302 (274), proline 286 (258), methionine 320 (292), serine 390 (362), threonine 404 (376), phenylalanine 336 (308), aspartic acid 418 (390), glutamic acid 432 (272), lysine 300 (168), arginine 340 (199), histidine 338 (197), tyrosine 466 (438), cystine 348 (106). Chromatographic peak area of quantitative ions was collected and integrated for quantification.^60^ The resultant data were calculated based on standard curves and normalized to internal standards to obtain their abundances in samples.

Peak areas of AMP, ADP and ATP at 346.0558 m/z, 426.0221 m/z and 505.9885 m/z were collected to quantify their abundances, respectively.

Quantifications of FTIR spectroscopy, nanoDSF fluorescence, ITC enthalpy change, glucose or sucrose contents for enzymic activity, and quantitative PCR were detailed in the method details section. Experimental replication was indicated in the Figure legends and in the method details section.

The statistical analysis was implemented using Microsoft Excel software for student’s-test, statistical difference was assigned when *p* values were <0.05 and significant difference when *p* values were <0.01.

## References

[1] Murata T. (1968). Enzymic mechanism of starch breakdown in germinating rice seeds I. An analytical study. Plant Physiol..

[2] Palmiano E.P., Juliano B.O. (1972). Biochemical changes in the rice grain during germination. Plant Physiol..

[3] Nomura T., Kono Y., Akazawa T. (1969). Enzymic mechanism of starch breakdown in germinating rice seeds II. scutellum as the site of sucrose synthesis. Plant Physiol..

[4] Humphreys T.E. (1987). Sucrose efflux and export from the maize scutellum. Plant Cell Environ..

[5] Sánchez-Linares L., Gavilanes-Ruiz M., Diaz-Pontones D., Guzman-Chavez F., Calzada-Alejo V., Zurita-Villegas V., Luna-Loaiza V., Moreno-Sanchez R., Bernal-Lugo I., Sanchez-Nieto S. (2012). Early carbon mobilization and radicle protrusion in maize germination. J. Exp. Bot..

[6] Hofstra G., Nelson C.D. (1969). The translocation of photosynthetically assimilated ^14^C in corn. Can. J. Bot..

[7] Gunning B.E. (1978). Age-related and origin-related control of the numbers of plasmodesmata in cell walls of developing Azolla roots. Planta.

[8] Rutschow H.L., Baskin T.I., Kramer E.M. (2011). Regulation of solute flux through plasmodesmata in the root meristem. Plant Physiol..

[9] Ji X., Van den Ende W., Van Laere A., Cheng S., Bennett J. (2005). Structure, evolution, and expression of the two invertase gene families of rice. J. Mol. Evol..

[10] Ruan Y.L. (2014). Sucrose metabolism: gateway to diverse carbon use and sugar signaling. Annu. Rev. Plant Biol..

[11] Sturm A., Hess D., Lee H., Lienhard S. (1999). Neutral invertase is a novel type of sucrose-cleaving enzyme. Physiol. Plant..

[12] Qi X., Wu Z., Li J., Mo X., Wu S., Chu J., Wu P. (2007). AtCYT-INV1, a neutral invertase, is involved in osmotic stress-induced inhibition on lateral root growth in Arabidopsis. Plant Mol. Biol..

[13] Vargas W.A., Pontis H.G., Salerno G.L. (2007). Differential expression of alkaline and neutral invertases in response to environmental stresses: characterization of an alkaline isoform as a stress-response enzyme in wheat leaves. Planta.

[14] Martín M.L., Lechner L., Zabaleta E.J., Salerno G.L. (2013). A mitochondrial alkaline/neutral invertase isoform (A/N-InvC) functions in developmental energy-demanding processes in Arabidopsis. Planta.

[15] Battaglia M.E., Martin M.V., Lechner L., Martínez-Noël G.M.A., Salerno G.L. (2017). The riddle of mitochondrial alkaline/neutral invertases: A novel Arabidopsis isoform mainly present in reproductive tissues and involved in root ROS production. PLoS One.

[16] Ruan Y.L., Jin Y., Yang Y.J., Li G.J., Boyer J.S. (2010). Sugar input, metabolism, and signaling mediated by invertase: roles in development, yield potential, and response to drought and heat. Mol. Plant.

[17] Vargas W., Cumino A., Salerno G.L. (2003). Cyanobacterial alkaline/neutral invertases. Origin of sucrose hydrolysis in the plant cytosol?. Planta.

[18] Yao S.-G., Taketa S., Ichii M. (2002). A novel short-root gene that affects specifically early root development in rice (Oryza sativa L.). Plant Sci..

[19] Jia L., Zhang B., Mao C., Li J., Wu Y., Wu P., Wu Z. (2008). OsCYT-INV1 for alkaline/neutral invertase is involved in root cell development and reproductivity in rice (Oryza sativa L.). Planta.

[20] Barratt D.H.P., Derbyshire P., Findlay K., Pike M., Wellner N., Lunn J., Feil R., Simpson C., Maule A.J., Smith A.M. (2009). Normal growth of Arabidopsis requires cytosolic invertase but not sucrose synthase. Proc. Natl. Acad. Sci. USA.

[21] Welham T., Pike J., Horst I., Flemetakis E., Katinakis P., Kaneko T., Sato S., Tabata S., Perry J., Parniske M., Wang T.L. (2009). A cytosolic invertase is required for normal growth and cell development in the model legume, Lotus japonicus. J. Exp. Bot..

[22] Jang J.C., León P., Zhou L., Sheen J. (1997). Hexokinase as a sugar sensor in higher plants. Plant Cell.

[23] Cho J.I., Ryoo N., Eom J.S., Lee D.W., Kim H.B., Jeong S.W., Lee Y.H., Kwon Y.K., Cho M.H., Bhoo S.H. (2009). Role of the rice hexokinases OsHXK5 and OsHXK6 as glucose sensors. Plant Physiol..

[24] Xiao W., Sheen J., Jang J.C. (2000). The role of hexokinase in plant sugar signal transduction and growth and development. Plant Mol. Biol..

[25] Xiong Y., McCormack M., Li L., Hall Q., Xiang C., Sheen J. (2013). Glucose-TOR signalling reprograms the transcriptome and activates meristems. Nature.

[26] Jang J.C., Sheen J. (1994). Sugar sensing in higher plants. Plant Cell.

[27] Levi C., Gibbs M. (1976). Starch degradation in isolated spinach chloroplasts. Plant Physiol..

[28] Chia T., Thorneycroft D., Chapple A., Messerli G., Chen J., Zeeman S.C., Smith S.M., Smith A.M. (2004). A cytosolic glucosyltransferase is required for conversion of starch to sucrose in Arabidopsis leaves at night. Plant J..

[29] Lu Y., Sharkey T.D. (2004). The role of amylomaltase in maltose metabolism in the cytosol of photosynthetic cells. Planta.

[30] Xie J., Cai K., Hu H.X., Jiang Y.L., Yang F., Hu P.F., Cao D.D., Li W.F., Chen Y., Zhou C.Z. (2016). Structural analysis of the catalytic mechanism and substrate specificity of Anabaena alkaline invertase InvA reveals a novel glucosidase. J. Biol. Chem..

[31] Aleshin A.E., Feng P.H., Honzatko R.B., Reilly P.J. (2003). Crystal structure and evolution of a prokaryotic glucoamylase. J. Mol. Biol..

[32] Kingsford-Adaboh R., Grosche M., Dittrich B., Luger P. (2000). DL-Arginine monohydrate at 100 K. Acta Cryst C.

[33] Zhang H., Zhou Y., Zhang G. (2010). Study on electronic structures and spectroscopic properties of arginine. J. Heilongjiang Inst. Sci. Technol..

[34] Li H., Wang Y., Ye M., Li S., Li D., Ren H., Wang M., Du L., Li H., Veglia G. (2020). Dynamical and allosteric regulation of photoprotection in light harvesting complex II. Sci. China. Chem..

[35] Li H., Yu Y., Ruan M., Jiao F., Chen H., Gao J., Weng Y., Bao Y. (2022). The mechanism for thermal-enhanced chaperone-like activity of α-crystallin against UV irradiation-induced aggregation of γD-crystallin. Biophys. J..

[36] Xiang L., Le Roy K., Bolouri-Moghaddam M.R., Vanhaecke M., Lammens W., Rolland F., Van den Ende W. (2011). Exploring the neutral invertase-oxidative stress defence connection in Arabidopsis thaliana. J. Exp. Bot..

[37] El-Brolosy M.A., Kontarakis Z., Rossi A., Kuenne C., Günther S., Fukuda N., Kikhi K., Boezio G.L.M., Takacs C.M., Lai S.L. (2019). Genetic compensation triggered by mutant mRNA degradation. Nature.

[38] Julius B.T., Leach K.A., Tran T.M., Mertz R.A., Braun D.M. (2017). Sugar transporters in plants: new insights and discoveries. Plant Cell Physiol..

[39] Li J., Kim Y.J., Zhang D. (2022). Source-to-sink transport of sugar and its role in male reproductive development. Genes.

[40] Gill R.A., Ahmar S., Ali B., Saleem M.H., Khan M.U., Zhou W., Liu S. (2021). The role of membrane transporters in plant growth and development, and abiotic stress tolerance. Int. J. Mol. Sci..

[41] Li Z., Wei X., Tong X., Zhao J., Liu X., Wang H., Tang L., Shu Y., Li G., Wang Y. (2022). The OsNAC23-Tre6P-SnRK1a feed-forward loop regulates sugar homeostasis and grain yield in rice. Mol. Plant.

[42] Zhao Z., Wang C., Yu X., Tian Y., Wang W., Zhang Y., Bai W., Yang N., Zhang T., Zheng H. (2022). Auxin regulates source-sink carbohydrate partitioning and reproductive organ development in rice. Proc. Natl. Acad. Sci. USA.

[43] Steeves T.A., Sussex I.M. (1989).

[44] Wang Z., Xie Y., Zhang H., Lin J., Liu Q., Wang T. (2010). Expression pattern of cell cycle genes suggests the developmental arrangement of vascular cells in sugarcane strand. Plant Growth Regul..

[45] Webster P.L., Langenauer H.D. (1973). Experimental control of the activity of the quiescent centre in excised root tips of Zea mays. Planta.

[46] Koch K. (2004). Sucrose metabolism: regulatory mechanisms and pivotal roles in sugar sensing and plant development. Curr. Opin. Plant Biol..

[47] Tognetti J.A., Pontis H.G., Martínez-Noël G.M.A. (2013). Sucrose signaling in plants: a world yet to be explored. Plant Signal. Behav..

[48] Yoon J., Cho L.H., Tun W., Jeon J.S., An G. (2021). Sucrose signaling in higher plants. Plant Sci..

[49] Tsai C.J., Del Sol A., Nussinov R. (2009). Protein allostery, signal transmission and dynamics: a classification scheme of allosteric mechanisms. Mol. Biosyst..

[50] Du X., Li Y., Xia Y.L., Ai S.M., Liang J., Sang P., Ji X.L., Liu S.Q. (2016). Insights into protein-ligand interactions: mechanisms, models, and methods. Int. J. Mol. Sci..

[51] Ross P.D., Subramanian S. (1981). Thermodynamics of protein association reactions: forces contributing to stability. Biochemistry.

[52] Perozzo R., Folkers G., Scapozza L. (2004). Thermodynamics of protein-ligand interactions: history, presence, and future aspects. J. Recept. Signal Transduct. Res..

[53] Araki T. (2001). Transition from vegetative to reproductive phase. Curr. Opin. Plant Biol..

[54] Counce P.A., Keisling T.C., Mitchell A.J. (2000). A uniform, objective, and adaptive system for expressing rice development. Crop Sci..

[55] Ohto M., Onai K., Furukawa Y., Aoki E., Araki T., Nakamura K. (2001). Effects of sugar on vegetative development and floral transition in Arabidopsis. Plant Physiol..

[56] Hirsche J., García Fernández J.M., Stabentheiner E., Großkinsky D.K., Roitsch T. (2017). Differential effects of carbohydrates on Arabidopsis pollen germination. Plant Cell Physiol..

[57] Yoshida S., Forno D.A., Cock J.H., Gomez K.A. (1976).

[58] Wang Z., Chen C., Xu Y., Jiang R., Han Y., Xu Z., Chong K. (2004). A practical vector for efficient knockdown of gene expression in rice (Oryza sativa L.). Plant Mol. Biol. Report..

[59] Warner C.A., Biedrzycki M.L., Jacobs S.S., Wisser R.J., Caplan J.L., Sherrier D.J. (2014). An optical clearing technique for plant tissues allowing deep imaging and compatible with fluorescence microscopy. Plant Physiol..

[60] Wang Z., Xue Z., Wang T. (2014). Differential analysis of proteomes and metabolomes reveals additively balanced networking for metabolism in maize heterosis. J. Proteome Res..

[61] Pryor K.D., Leiting B. (1997). High-level expression of soluble protein in Escherichia coli using a His6-tag and maltose-binding-protein double-affinity fusion system. Protein Expr. Purif..

[62] Li H., Ke H., Ren G., Qiu X., Weng Y.X., Wang C.C. (2009). Thermal-induced dissociation and unfolding of homodimeric DsbC revealed by temperature-jump time-resolved infrared spectra. Biophys. J..

[63] Zhang Y., Li B., Xu Y., Li H., Li S., Zhang D., Mao Z., Guo S., Yang C., Weng Y., Chong K. (2013). The cyclophilin CYP20-2 modulates the conformation of BRASSINAZOLE-RESISTANT1, which binds the promoter of FLOWERING LOCUS D to regulate flowering in Arabidopsis. Plant Cell.

[64] Königshofer H., Loppert H.G. (2015). Regulation of invertase activity in different root zones of wheat (Triticum aestivum L.) seedlings in the course of osmotic adjustment under water deficit conditions. J. Plant Physiol..

[65] Ishimaru T., Hirose T., Matsuda T., Goto A., Takahashi K., Sasaki H., Terao T., Ishii R.i., Ohsugi R., Yamagishi T. (2005). Expression patterns of genes encoding carbohydrate-metabolizing enzymes and their relationship to grain filling in rice (Oryza sativa L.): comparison of caryopses located at different positions in a panicle. Plant Cell Physiol..

