# Supplementary material for: A neutral invertase controls cell division besides hydrolysis of sucrose for nutrition during germination and seed setting in rice
Source: iScience. 2024 Jun 8;27(7):110217. doi: 10.1016/j.isci.2024.110217 (PMC11237924; doi:10.1016/j.isci.2024.110217)
Supplement: Document S1. Figures S1–S11 and Table S1–S8 [file mmc1.pdf]

iScience, Volume *27*

Supplemental information

A neutral invertase controls cell division besides hydrolysis of sucrose for nutrition during germination and seed setting in rice

Zizhang Wang, Hao Li, and Yuxiang Weng

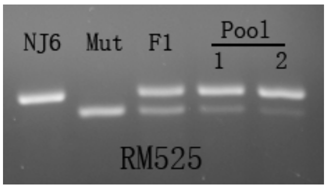

Figure S1 (Related to Figure 1). Rough mapping locates the mutated gene near marker RM525 on Chr. 2

NJ6, male parent; Mut, mutant, female parent; F1, first filial generation; Pool 1 and pool 2 each, fifteen DNA samples from random seedling with short root phenotype were quantified and equally mixed for PCR template, showing the band of female parent were weaker than that in F1.

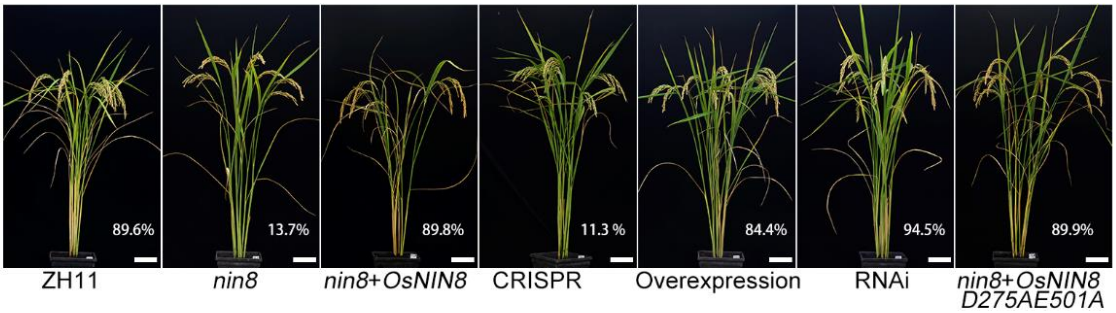

Figure S2 (Related to Figure 1 and Figure 4). Phenotype of genetical modification of *OsNIN8* lines at mature stage

nin8+ *OsNIN8*, complementation of mutated gene *OsNIN8m* with wild-type *OsNIN8* gene in *nin8*; CRISPR, knockout of *OsNIN8* using CRISPR/Cas9 method; Overexpression, overexpression of *OsNIN8* in ZH11; RNAi, knockdown of *OsNIN8* in ZH11; *nin8*+ *OsNIN8D275AE501A*, complementation of *OsNIN8m* with *OsNIN8D275AE501A*, which both D275 and E501 of OsNIN8 had been mutated into A, by transformation of the construction in *nin8*. Minor panicle hood of *nin8* and CRISPR plants indicated their low seed setting rate, seed set rate was provided alongside. Bar=10 cm.

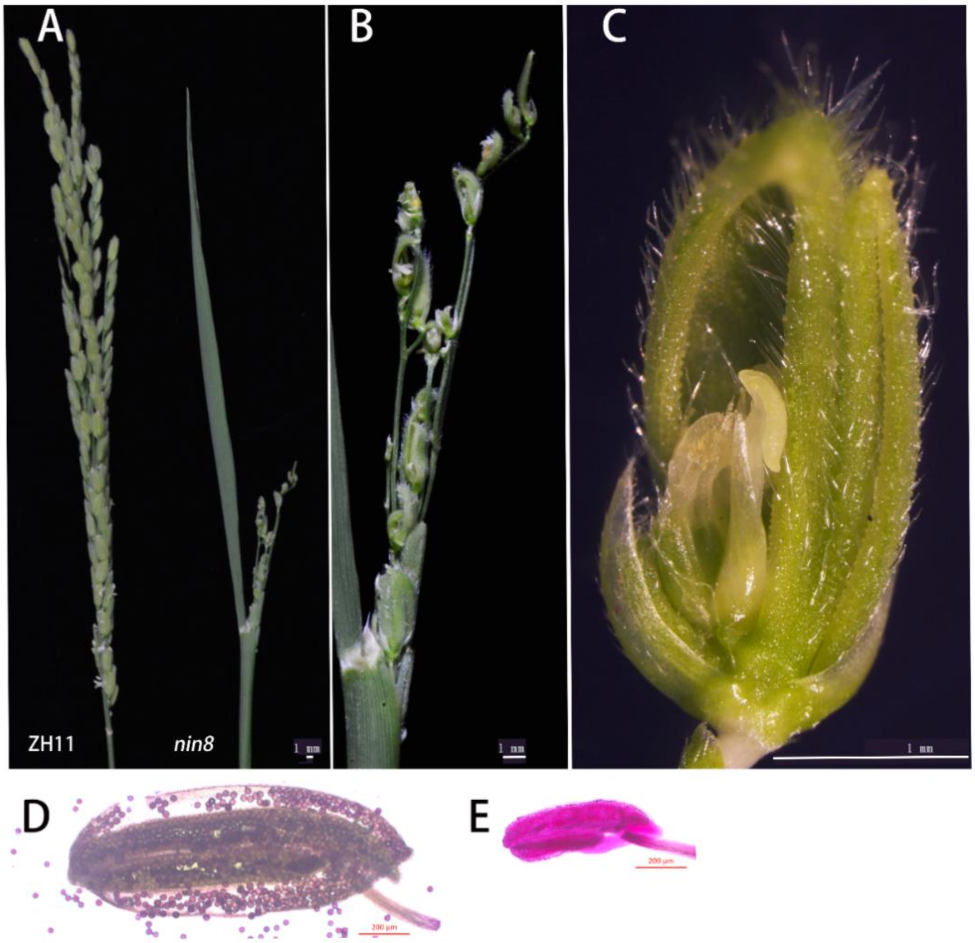

Figure S3 (Related to Figure 1). Sensitive floret and pollen developments in *nin8* cultivated in cool condition

(A) Spike of ZH11 and *nin8* at flowering stage (cultural temperature was about 24℃). Bar=1 mm;

(B) Magnified image of *nin8* spike. Bar=1 mm;

(C) A floral organ of *nin8* showing aplasia in glume, stamen and pistil. Bar=1 mm;

(D) Anther and pollen of ZH11 by Alexander staining showing normal pollen grains. Bar=200 μm;

(E) Anther of *nin8* by Alexander staining showing maldevelopment without pollens. Bar=200 μm.

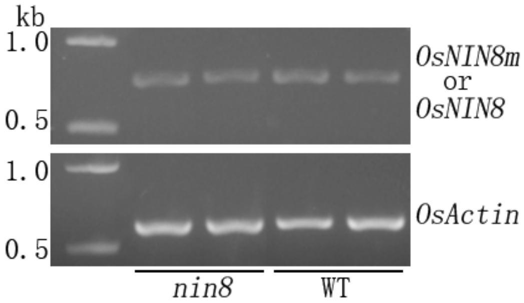

Figure S4 (Related to Figure 1). Expression of *OsNIN8m* or *OsNIN8* in roots at 17 DAI in *nin8* or WT using RT-PCR

OsActin was used as internal standard. *OsNIN8m*, the mutated *OsNIN8* in *nin8* mutant.

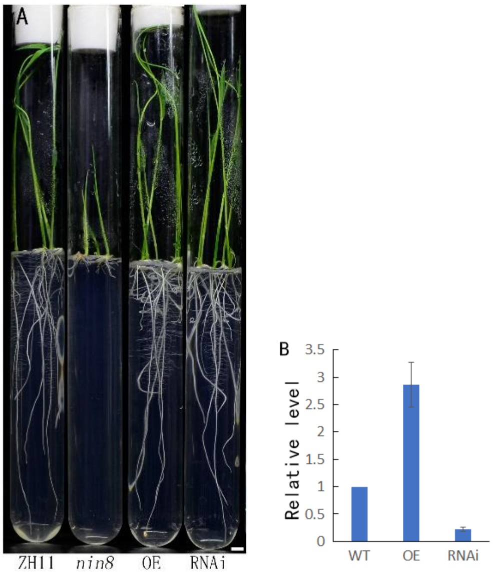

Figure S5 (Related to Figure 1). Phenotypes of overexpression (OE) and RNAi of *OsNIN8* at seedling stage

ZH11, wild-type (WT); *nin8*, mutant; OE, *OsNIN8* was droved by UBI promoter and transformed in ZH11; RNAi, *OsNIN8* was performed RNAi construction and transformed in ZH11.

(A) Sterile culture of seeds was carried out in test-tube for about 2 weeks. bar=6 mm;

(B) Relative expression levels of *OsNIN8* in root among ZH11, OE and RNAi lines. Roots were collected and analyzed *OsNIN8* transcriptional expression using real-time PCR. Level in WT was normalized to 1.

Figure S6 (Related to Figure 1). Functional separation of OsNIN8 from OsNIN1-7

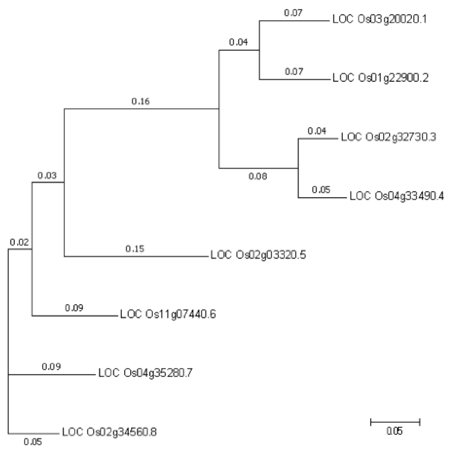

(A) Phylogenetic analysis of 8 alkaline/neutral invertases in rice

A conserved region about 455 AA, which are most full-length each, of 8 rice neutral/alkaline invertases were complete alignment using ClustalX 2.1. The NJ tree was generated by using MEGA 7.0. Numbers 1-8 suffixed gene ID, serial number of 8 alkaline/neutral invertases; OsNIN8 (LOC_Os02g34560.8) showed no branches and separation from other neutral/alkaline invertases.

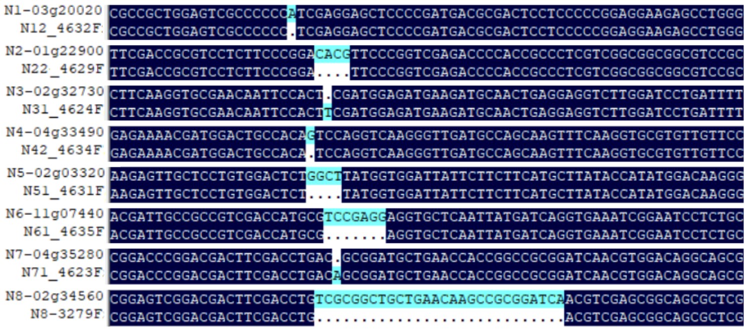

(B) Genomic sequences of 8 neutral/alkaline invertases after CRISPR/Cas9 modification aligned to respective WT sequence.

N1-N8, neutral/alkaline invertase 1-8; Gaps, insertion or deletion led to frameshift mutation.

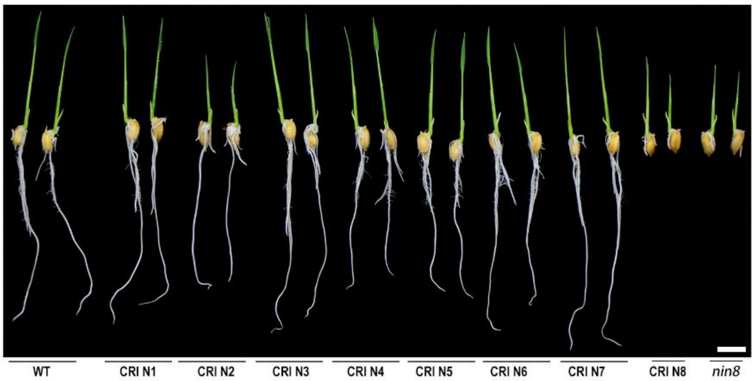

(C) Phenotypes of CRISPR/Cas9 modification lines at DAI 10

CRI N1-8, CRISPR/Cas9 modification of neutral/alkaline invertase1-8; Bar=1 cm.

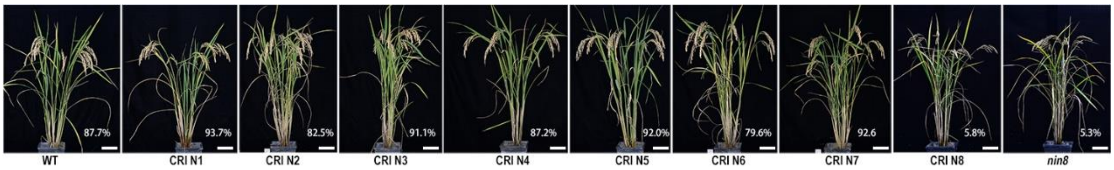

(D) Phenotypes of CRISPR/Cas9 modification lines at mature stage

CRI N1-8, CRISPR/Cas9 modification of neutral/alkaline invertase1-8; Low degree of panicle hood in CRI N8 and *nin8* indicated their low seed setting rate, Seed set rate was provided alongside. Bar=10 cm.

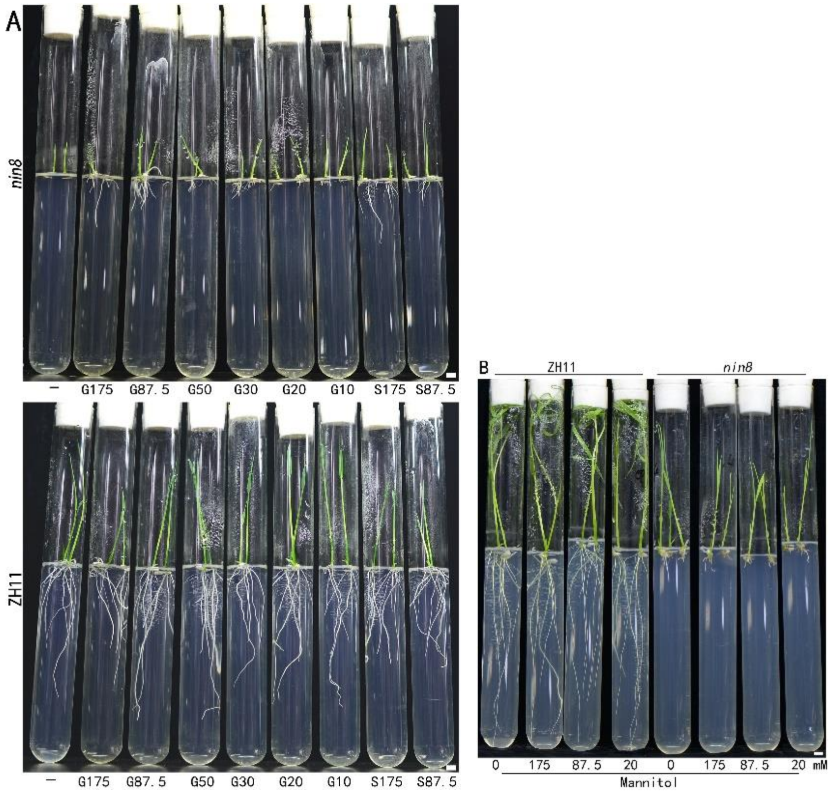

Figure S7 (Related to Table 1). The phenotype of retarded radicle in *nin8* was not recovered by adding glucose or sucrose to media during germination

(A) Seeds of ZH11 or *nin8* are inoculated on Yoshida media without (-) or with glucose (G) or sucrose (S) at different concentrations (mM) for 10 days; Bar=6 mm;

(B) Seeds of ZH11 or *nin8* are inoculated on Yoshida media without (0) or with mannitol of 175, 85.7 or 20 mM for 20 days to assess osmotic effect; Bar=6 mm.

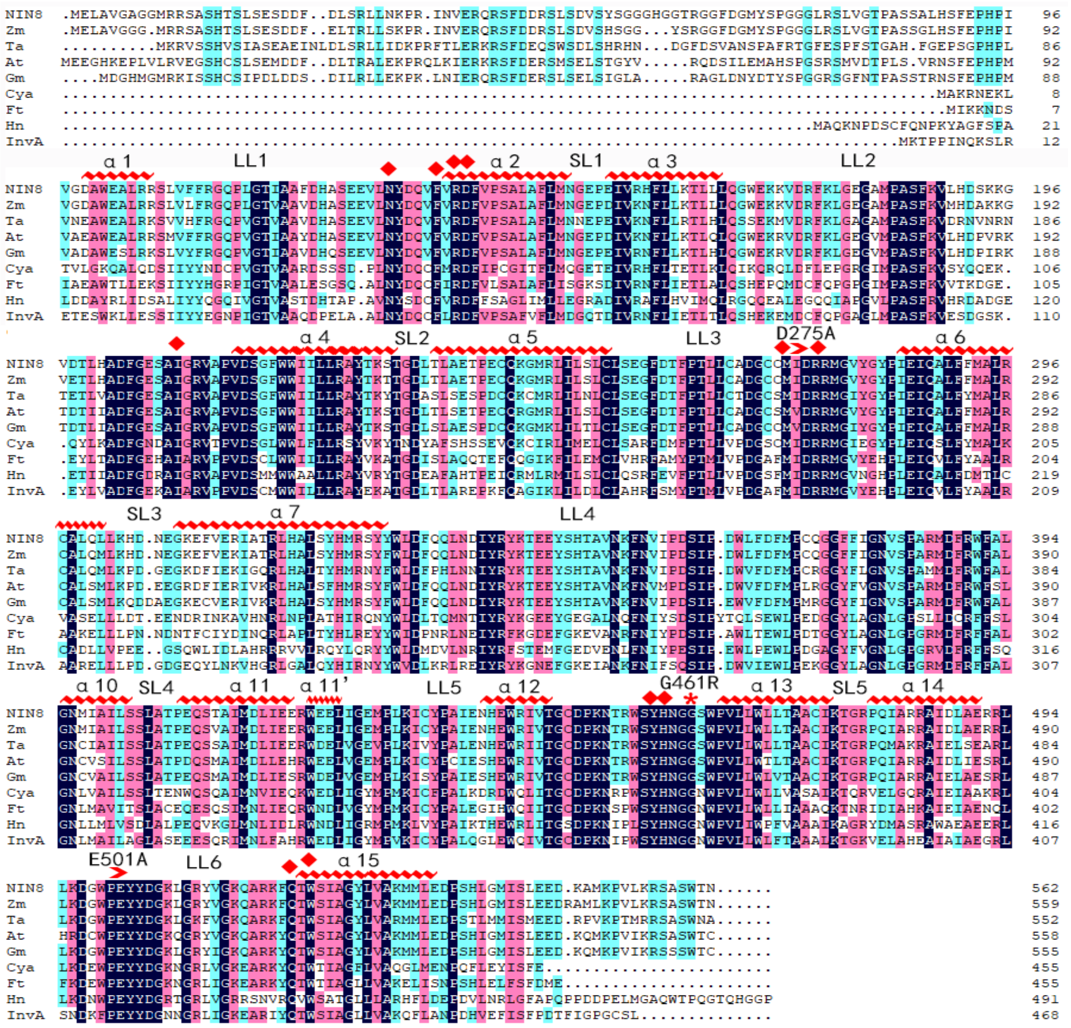

Figure S8 (Related to Figure 3). Alignment of sequence of neutral/alkaline invertases from different species

NIN8, OsNIN8; Zm, *Zea mays*; Ta, *Triticum aestivum*; At, *Arabidopsis thaliana*; Gm, *Glycine max*; Cya, *Cyanothece*; Ft, *Fischerella thermalis*; Hn, *Halothiobacillus neapolitanus*; InvA, *Anabaena azollae* neutral/alkaline invertase. Wave line, α helix; Diamond, binding residue; Arrowhead, catalytic residue; Asterisk, mutated residue.

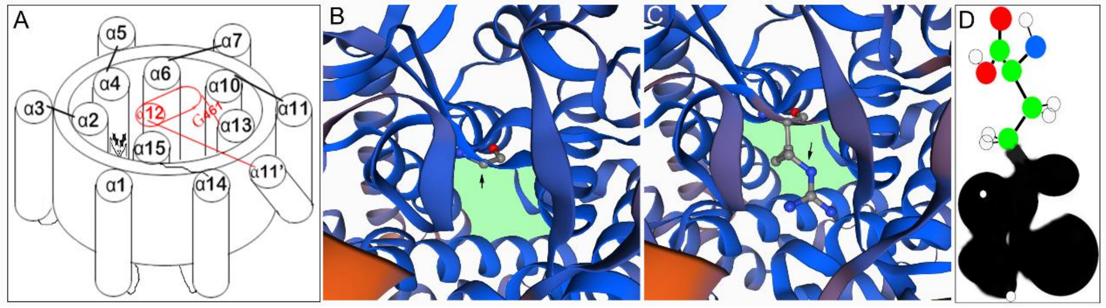

Figure S9 (Related to Figure 3). Mutation of Gly[461]Arg changes secondary structure of OsNIN8m

(A) Diagram of the OsNIN8 $(\alpha/\alpha)_6$ barrel according to alignment from a *Thermoanaerobacterium thermosaccharolyticum* glucoamylase (S1) and an *Anabaena* alkaline invertase (S2). Six helixes (α2, α4, α6, α10, α13 and α15) forming the inside wall, and other six helixes (α1, α3, α5, α7, α11 and α14) forming the outside wall of the barrel, G461R between α12 and α13 above the open of the barrel, and some stars in the barrel standing for sucrose molecules were indicated;

(B-C) Inside α_6 barrel of OsNIN8 and OsNIN8m matching the *Anabaena* alkaline invertase by homology modeling (SWISS-MODEL). Glycine residue (ball-and-stick structure, black arrowhead) allowed the mouth of OsNIN8 open but arginine residue (black arrowhead) led the mouth of OsNIN8m to be blocked. Openings of inside wall of the barrel were indicated in light green. Ball-and-stick in grey, carbon atom; in blue, nitrogen atom; in red, oxygen atom;

(D) Molecular structure of arginine. Frontier orbital electron cloud in black; ball in green, carbon atom; in blue, nitrogen atom; in red, oxygen atom; in white, hydrogen atom.

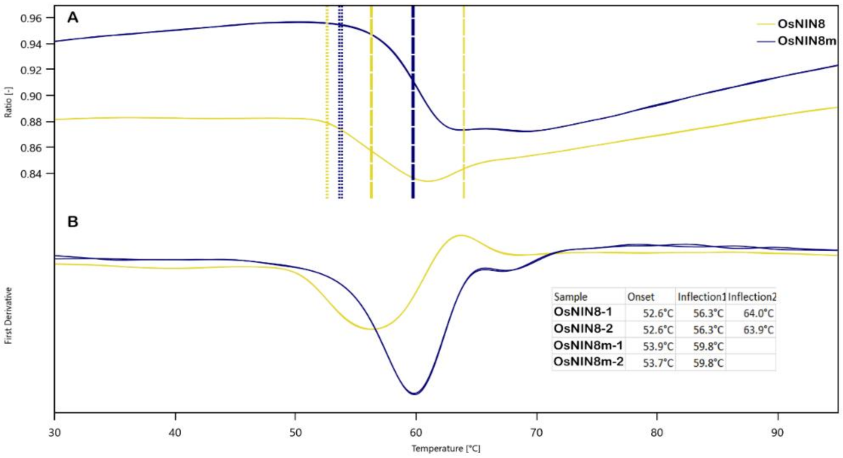

Figure S10 (Related to Figure 3). Thermal stability of OsNIN8 and OsNIN8m presented by ratio of integrated fluorescence of 350nm/330nm (n=2)

(A) There were two inflection points on OsNIN8 but only one inflection point on OsNIN8m. The start and the end temperatures of inflection point were indicated with lines of dashes;

(B) First derivative of the ratio to determine their melting temperatures. Temperatures of onset, inflection points 1 and 2 shown.

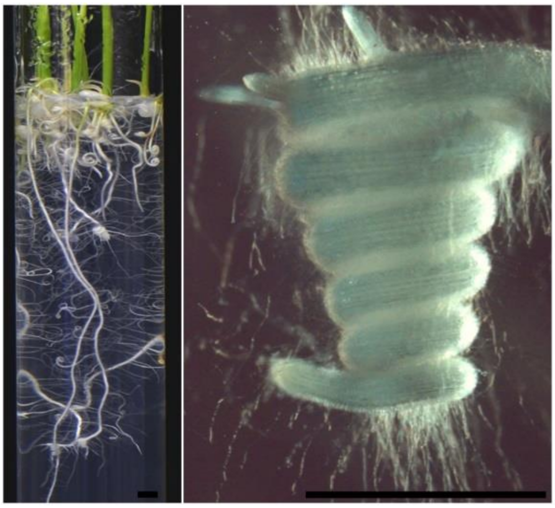

Figure S11 (Related to Figure 2). Root growing toward walls curls into helical
More seeds inoculated in a small test tube was prone to helical root, diameter of the test tube was 1.5 cm; Bar=1.5 mm.

Table S1 (Related to Figure 1). Primer sequence of markers for fine mapping

Marker	Forward primer	Reverse primer
RM6509	GGTGTTTTGTGGTGTTGTGC	CTCGAACTGCGAGTAGGACC
RM2634	GATTGAAAATTAGAGTTTGCAC	TGCCGAGATTTAGTCAACTA
398	GGAAGAGGAAGGTCGGTGATGG	CGGCGAACATGTCGTTCATCC
402	GGGCATGCTCTACCACTCTTACC	CTCACCAAAGATTCGGTATGTGC
403	GTGCTCCTCTCCGTGCTATGC	TACGTAATGCGGTGCTCCTTGC
404	CATAGGCACACCCACTCG	AATTCAACGGAACAGAACTGTC
242	ATTCTTCCTCGTGAGGTGA	CATGAGCAATAGTACAGGACAAGG
252	ATTAACAATGGACCCACAAC	CAAGCTGATGTCCCTGTC
262	CATTCCGTCTCGGCTCAACT	CAGAGCAAGGTGGCTTGC
666	GAAGGTGGAAACTGTTGTTCAGG	GACAGTTTAGCCTGTACTCCATCC
RM1303	CTGATCTTGGTGAGCGAGTG	TACGGATCAGCACTCAGCAC
RM3688	GTTGAATCAAGCTGTGCAGC	AGCTAGGCAAAGCATGCATG

Table S2 (Related to Table 1). Sugars and citric acid contents in roots of ZH11 and *nin8* at DAI 17 (μg/gFW)

		Suc	Mal	Glc	Fru	Cit
nin8	Mean	40.25	2.84	1443.94	1454.13	169.65
	SEM	7.01	0.11	101.46	71.92	1.11
ZH11	Mean	1.09	0.06	4.16	41.49	5.48
	SEM	1.15	0.10	1.11	7.80	0.53
	Fold	36.96**	50.00**	347.12**	35.05**	30.95**

Roots of ZH11 and *nin8* at DAI 17 (foliar age 2.5) were collected and sugars and citric acid were determined involved use of GC-TOF-MS method (data were from 3 independent experiments). Suc, sucrose; Mal, maltose; Glc, glucose; Fru, fructose; Cit, citric acid. SEM, standard error of mean; Fold, ratio of mean of *nin8* to ZH11; Fold in red indicated the ratio was greater than 1. ** indicated statistically significant difference; DAI, day after inoculation.

Table S3 (Related to Table 1). Content of free amino acids in roots of ZH11 and *nin8* at DAI 17 (μg/gFW)

		Ala	Val	Leu	Ile	Pro	Gly	Ser	Thr	Asp	Met	Glu	Phe	Lys	Tyr
nin8	Mean	137.15	21.60	18.07	11.70	5.89	3.35	42.77	14.13	69.41	4.05	35.54	11.01	19.62	6.08
	SEM	14.22	1.49	3.40	1.62	0.53	0.15	3.84	0.70	3.42	0.03	2.70	1.36	1.79	0.36
ZH11	Mean	13.44	3.69	2.48	1.90	0.61	0.43	4.90	2.80	8.05	1.94	39.42	3.68	5.00	0.42
	SEM	1.23	0.09	0.32	0.03	0.01	0.00	0.22	0.22	1.04	0.35	3.65	0.46	0.19	0.71
	Fold	10.20**	5.85**	7.29**	6.15**	9.58**	7.77**	8.72**	5.05**	8.62**	2.09**	0.90*	2.99**	3.92**	14.45**

Roots of ZH11 and *nin8* at DAI 17 were collected and 14 free amino acids were determined involved use of GC-QqQ-MS/MS method (data were from 3 independent experiments). Ala, alanine; Val, valine; Leu, leucine; Ile, isoleucine; Pro, proline; Gly, glycine; Ser, serine; Thr, threonine; Asp, aspartic acid; Met, methionine; Glu, glutamic acid; Phe, phenylalanine; Lys, lysine; Tyr, tyrosine. SEM, standard error of mean; Fold, ratio of mean of *nin8* to ZH11; Fold in red indicated the ratio was greater than 1, in blue was smaller than 1. ** indicated statistically significant difference; * was statistical difference. DAI, day after inoculation.

Table S4 (Related to Table 1). Sugars and citric acid contents in endosperm of ZH11 and *nin8* at DAI 10 and 17 (µg/gFW)

		Suc	Mal	Glc	Fru	Cit
DAI 10						
nin8	Mean	4317.19	6665.28	5046.83	1428.74	86.72
	SEM	23.89	491.73	659.01	108.60	10.73
**ZH11**	Mean	1976.14	10.74	721.57	234.49	41.32
	SEM	282.63	0.54	50.23	27.04	4.03
	Fold	2.18**	620.88**	6.99**	6.09**	2.10**
DAI 17						
nin8	Mean	3782.13	2450.66	4389.94	1771.81	94.19
	SEM	505.34	107.00	300.31	55.65	2.89
**ZH11**	Mean	3.58	0.54	3.10	13.05	12.46
	SEM	0.47	0.93	0.87	2.86	1.21
	Fold	1055.81**	4548.09**	1415.67**	135.75**	7.56**

Notes see Table S2.

Table S5 (Related to Table 1). Content of free amino acids in endosperm of ZH11 and *nin8* at DAI 10 and 17 (µg/gFW)

		Ala	Val	Leu	Ile	Pro	Gly	Ser	Thr	Asp	Met	Glu	Phe	Lys	Tyr
DAI 10															
nin8	Mean	64.64	105.10	102.26	60.32	24.51	6.22	97.69	32.24	51.86	20.71	57.28	69.29	112.47	108.48
	SEM	2.32	3.93	3.97	2.78	0.89	0.16	15.65	2.77	6.54	1.74	1.30	8.68	14.16	10.10
**ZH11**	Mean	17.02	14.87	17.01	8.71	3.27	2.20	15.46	10.35	9.28	4.90	32.93	9.97	22.11	5.31
	SEM	0.81	0.40	2.18	0.38	0.08	0.07	0.06	0.43	0.32	0.40	1.26	0.38	0.91	0.19
	Fold	3.80**	7.07**	6.01**	6.92**	7.50**	2.83**	6.32**	3.11**	5.59**	4.23**	1.74**	6.95**	5.09**	20.43**
DAI 17															
nin8	Mean	42.43	37.31	38.82	20.79	9.29	3.98	44.94	21.26	33.07	10.31	37.66	33.14	73.82	44.61
	SEM	2.55	2.49	5.63	2.46	0.48	0.11	3.97	0.98	2.02	0.31	5.85	2.00	2.19	1.31
**ZH11**	Mean	7.37	3.26	1.95	1.32	0.65	0.31	2.11	3.04	3.18	2.19	16.21	3.87	6.20	0.00
	SEM	0.34	0.10	0.21	0.01	0.03	0.00	0.12	0.08	0.26	0.05	1.54	0.16	0.40	0.00
	Fold	5.76**	11.44**	19.94**	15.70**	14.34**	13.00**	21.34**	7.00**	10.40**	4.72**	2.32**	8.57**	11.90**	**

Notes see Table S3.

Table S6 (Related to Table 1). Sugars and citric acid contents in leaf of ZH11 and *nin8* at DAI 10 and 17 (μg/gFW)

		Suc	Mal	Glc	Fru	Cit
DAI 10						
nin8	Mean	873.30	33.12	1214.70	1686.81	95.52
	SEM	48.57	3.49	27.90	30.00	1.52
**ZH11**	Mean	35.74	2.58	579.53	309.13	164.14
	SEM	5.38	2.33	122.07	28.65	8.01
	Fold	24.43**	12.82**	2.10**	5.46**	0.58**
DAI 17						
nin8	Mean	854.25	15.01	1147.02	974.97	90.59
	SEM	121.87	2.41	37.67	37.99	8.77
**ZH11**	Mean	56.92	0.24	455.51	285.21	18.87
	SEM	7.66	0.41	22.19	33.78	0.58
	Fold	15.01**	63.75**	2.52**	3.42**	4.80**

Notes see Tables S2 and S3.

Table S7 (Related to Table 1). Content of free amino acids in leaf of ZH11 and *nin8* at DAI 10 and 17 (μg/gFW)

		Ala	Val	Leu	Ile	Pro	Gly	Ser	Thr	Asp	Met	Glu	Phe	Lys	Tyr
DAI 10															
nin8	Mean	89.51	46.92	13.75	16.88	10.44	4.45	143.69	32.85	80.81	10.08	70.79	10.66	18.72	5.22
	SEM	10.16	1.98	2.77	1.46	0.18	0.21	9.02	0.89	5.62	0.60	6.51	0.97	2.13	0.51
**ZH11**	Mean	60.14	15.44	5.00	6.49	3.91	1.33	27.43	12.20	117.19	2.39	76.12	9.44	13.78	4.36
	SEM	3.05	1.25	1.78	0.26	0.05	0.04	0.30	1.00	2.72	0.21	4.79	0.99	0.50	0.15
	Fold	1.49**	3.04**	2.75**	2.60**	2.67**	3.34**	5.24**	2.69**	0.69**	4.22**	0.93	1.13	1.36	1.20
DAI 17															
nin8	Mean	133.71	14.75	5.58	5.32	4.58	2.16	38.62	14.57	118.07	4.85	54.51	8.41	19.40	5.39
	SEM	13.74	0.48	2.46	0.71	0.34	0.18	2.25	0.57	6.58	0.61	6.98	0.58	1.71	0.32
**ZH11**	Mean	30.46	40.48	10.20	27.07	5.24	2.53	25.81	23.94	31.24	7.49	46.06	65.13	19.05	10.03
	SEM	3.34	5.93	2.46	4.73	0.30	0.02	1.31	0.21	1.05	0.72	3.89	4.06	2.88	0.66
	Fold	4.39**	0.36**	0.55*	0.20**	0.87	0.85	1.50**	0.61*	3.78**	0.65*	1.18	0.13**	1.02	0.54**

Notes see Table S3.

Table S8 (Related to Figure 5). Primer sequences for Q-PCR

Gene	Primer	Sequence of primer
Ubiquitin-conjugating enzyme	UBIF	ACTCTCAATTTGCTCGCTGTTG
	UBIR	AATCTCTGGAACCAATGGATCATC
Sucrose synthase	SUS1F	AGCCATGACATGTGGTTTGC
	SUS1R	ATCTCAGCAGGGCCACCAT
	SUS2F	GAAACCCGCCGCTACCTT
	SUS2R	GCTAGCCATGGTGCGGTACT
Invertase inhibitor	INH1F	CGTCTGCTACGACTCCC
	INH1R	CGTGGCTGGTCTGGAAC
	INH2F	CCGCTGGTTGCGGAGTAC
	INH2R	CTATCAATGCGAGAGCAATGGA
Sucrose transporter	SUT1F	GGTGACCCAAAGGGAACTGAT
	SUT1R	TGCCCTGACACCCTGGTT
	SUT2F	CACCGAGAATGACCCAAAGAG
	SUT2R	AGGGCCATGAACAATGAGAAGT
Neutral/alkaline invertase	NIN1F	CCATGGGCATCCTCTTGAGA
	NIN1R	GGGCACAACGCAAAGCA
	NIN2F	GGCTGTGCCTCTTGATGACA
	NIN2R	TCTCCAAAGTCAGGGTCCAAA
	NIN3F	AGTCCGGTCGGGACGATT
	NIN3R	ATAATTCATCGGATTGGCATCAT
	NIN4F	AAGGGTTGATGCCAGCAAGT
	NIN4R	CATCATCGTCCCCGTCAAGT
	NIN5F	TTCTGCCTGGGCAACTTCA
	NIN5R	GCCTGTTCTCCGGTTGTCA
	NIN6F	TTTGCCCTTGGAAATTGCA
	NIN6R	TGCCACTGATTGCTCTGGAGTA
	NIN7F	TGCCGCTCAAGATCAGCTT
	NIN7R	GCACCCGGTGACGAACTC
	NIN8F	CGCCGGAATGCCAGAAG
	NIN8R	ATCAAACCCCTCAGACAAGCA
Vacuolar invertase	VIN1F	CGTCGGCAAAGCAACACA
	VIN1R	CCGAAGTGTCCACCTCGAA
	VIN2F	GCGCACCGGATTCCATT
	VIN2R	CGTTGGGATCGTTCATCCA

Table S8 (Related to Figure 5). Primer sequences for Q-PCR (continue)

Gene	Primer	Sequence of primer
Cell wall invertase	CWIN1F	ATTCCGCGATCCGACAAC
	CWIN1R	AACGAGCATCCGCCAATG
	CWIN2F	CGTTTGCAGGGTTCGTTGA
	CWIN2R	CGATCAGGCTCCTCAGAGATATC
	CWIN3F	CATGTGCAACGACCCTACCA
	CWIN3R	GCGAAGGTCGGCCTGTAGA
	CWIN4F	CGGGTACACGGTCCTCATGT
	CWIN4R	GTACACCCCTGCTCTTGAAGTTG
	CWIN5F	CGGCGTGCAGACATTCC
	CWIN5R	CAGCTGCTTGCCGTCCTT
	CWIN6F	GCCGCCCATGATGAACTG
	CWIN6R	GCGGAGGCAATGCACAAT

Supplemental references

(S1) Aleshin, A.E., Feng, P.H., Honzatko, R.B., and Reilly, P.J. (2003). Crystal structure and evolution of a prokaryotic glucoamylase. J. Mol. Biol. *327*, 61-73. DOI: https://doi.org/10.1016/s0022-2836(03)00084-6.

(S2) Xie, J., Cai, K., Hu, H.X., Jiang, Y.L., Yang, F., Hu, P.F., Cao, D.D., Li, W.F., Chen, Y., and Zhou, C.Z. (2016). Structural analysis of the catalytic mechanism and substrate specificity of Anabaena alkaline invertase InvA reveals a novel glucosidase. J. Biol. Chem. *291*, 25667-25677. DOI: https://doi.org/10.1074/jbc.M116.759290.
